# Externalized phosphatidylinositides on apoptotic cells are eat-me signals recognized by CD14

**DOI:** 10.1038/s41418-022-00931-2

**Published:** 2022-01-11

**Authors:** Ok-Hee Kim, Geun-Hyung Kang, June Hur, Jinwook Lee, YunJae Jung, In-Sun Hong, Hookeun Lee, Seung-Yong Seo, Dae Ho Lee, Cheol Soon Lee, In-Kyu Lee, Susan Bonner-Weir, Jongsoon Lee, Young Joo Park, Hyeonjin Kim, Steven E. Shoelson, Byung-Chul Oh

**Affiliations:** 1 Department of Physiology, Lee Gil Ya Cancer and Diabetes Institute, Gachon University College of Medicine, Incheon, 21999 Republic of Korea; 2 Department of Microbiology, Lee Gil Ya Cancer and Diabetes Institute, Gachon University College of Medicine, Incheon, 21999 Republic of Korea; 3 Department of Molecular Medicine, Lee Gil Ya Cancer and Diabetes Institute, Gachon University College of Medicine, Incheon, 21999 Republic of Korea; 4 College of Pharmacy, Gachon University, Incheon, 21936 Republic of Korea; 5 Department of Internal Medicine, Gachon University Gil Medical Center, Incheon, 21565 Republic of Korea; 6 Medical Health Research Center, Korea University College of Medicine, Seoul, 02841 Republic of Korea; 7 Department of Biomedical Science, Graduate School of Medicine, Kyungpook National University, Daegu, 41404 Republic of Korea; 8 Joslin Diabetes Center and Department of Medicine, Harvard Medical School, One Joslin Place, Boston, MA 02215 USA; 9 Soonchunhyang Institute of Medi-bio Science (SIMS), Cheonan-si, 31151 Republic of Korea; 10 Department of Internal Medicine, Seoul National University Hosptial, Seoul, 03080 Republic of Korea; 11 Department of Radiology, Seoul National University College of Medicine, Seoul, 03080 Republic of Korea

**Keywords:** Phospholipids, Cell death and immune response

## Abstract

Apoptotic cells are rapidly engulfed and removed by phagocytes after displaying cell surface eat-me signals. Among many phospholipids, only phosphatidylserine (PS) is known to act as an eat-me signal on apoptotic cells. Using unbiased proteomics, we identified externalized phosphatidylinositides (PIPs) as apoptotic eat-me signals recognized by CD14^+^ phagocytes. Exofacial PIPs on the surfaces of early and late-apoptotic cells were observed in patches and blebs using anti-PI(3,4,5)P_3_ antibody, AKT- and PLCδ PH-domains, and CD14 protein. Phagocytosis of apoptotic cells was blocked either by masking exofacial PIPs or by CD14 knockout in phagocytes. We further confirmed that exofacial PIP^+^ thymocytes increased dramatically after in vivo irradiation and that exofacial PIP^+^ cells represented more significant populations in tissues of *Cd14*^−/−^ than WT mice, especially after induction of apoptosis. Our findings reveal exofacial PIPs to be previously unknown cell death signals recognized by CD14^+^ phagocytes.

## Introduction

Apoptotic cells initiate their own clearance by displaying cell surface eat-me signals which are recognized by tissue phagocytes [1, 2]. The rapid removal of apoptotic cells avoids potential inflammatory responses and the release of cellular contents, which damages tissues and promotes autoimmunity. Many eat-me signals have been identified, including phosphatidylserine (PS), oxidised lipids, intercellular adhesion molecule-3, and calreticulin [3, 4]. Externalized PS is a well-established eat-me signal for the phagocytic clearance of apoptotic cells [1, 2, 5–8]. Over 20 distinct phagocyte proteins of various structural classes are associated with PS-mediated recognition and clearance [2, 9]. This suggests that regions of the plasma membrane lose phospholipid polarity as cells undergo apoptosis, which is otherwise maintained with high fidelity through energy-dependent transporters and flippases [10–12].

PS, phosphatidylethanolamine (PE), and phosphatidylinositides (PIPs) are normally restricted to the cytoplasmic leaflet of the plasma membrane, whereas phosphatidylcholine (PC) and sphingomyelin are more prevalent in the outer leaflet. This asymmetric distribution of phospholipids in the inner and outer leaflets of the bilayer influences membrane biophysical properties and plays an essential role in cell signaling and regulation [11, 13]. Perturbations in phospholipid asymmetry are uncommon, with the noted exception of externalized PS serving as an apoptotic cell eat-me signal to trigger cell removal by phagocytes via efferocytosis [1, 2, 5, 6, 9]. PIPs have small but highly dynamic intracellular concentrations and regulate a wide variety of intracellular processes, including vesicle transport, cytoskeletal reorganization, and signal transduction [14–16]. However, neither the existence of externalized PIPs nor their physiological functions have been previously described.

CD14 binds and chaperones bacterial lipopolysaccharide (LPS) to Toll-like receptor-4 (TLR4) at the cell surface [17]. CD14 is also known as a co-receptor for recognition and endocytosis of bacterial LPS along with TLR4 [18] and myeloid differentiation factor 2 (MD-2) [19, 20]. Apoptotic cells have been shown to accumulate in the tissues of *Cd14*^−/−^ mice [21], suggesting that CD14 may function in the clearance of apoptotic cells as well as bacteria. Previous studies have also shown that CD14 mediates the cellular uptake and metabolism of extracellular PIPs [22], although the potential apoptotic eat-me signals recognized by CD14 remain unknown [21, 23, 24]. These results suggest that CD14 could potentially interact with externalized cellular PIPs as eat-me signals for recognizing apoptotic cells.

Our previous studies suggested that iron-calcium inositol hexakisphosphate (IC-IP_6_) is the phagocytic target for macrophages in vivo [25, 26]. Therefore, we performed unbiased IP_6_-based affinity proteomics to investigate how macrophages preferentially engulf IC-IP_6_. We found that CD14^+^ macrophages specifically bind and take up IC-IP_6_, which mimics the headgroup of PIPs. We hypothesized that CD14^+^ phagocytes would recognize and engulf apoptotic cells that displayed externalized PIPs on their surfaces in response to apoptotic stimuli. We found that externalized PIPs on the apoptotic cell surface could be an eat-me signal recognized by CD14^+^ phagocytes. Collectively, we showed that PIP_3_ has two opposing signaling roles in determining cell fate; flipped PIP_3_ is a cell death signal recognized by phagocytes, whereas inward-facing PIP_3_ elicits survival signals for cell growth [27, 28].

## Materials and methods

### Animal care and use

WT and *Cd14*^−/−^ C57Bl/6 mice were obtained from the Jackson Laboratory. Male mice were studied under protocols approved by the Animal Ethics Committee of Gachon University, Lee Gil Ya Cancer and Diabetes Institute (LCDI20130062, LCDI20160030, and LCDI20210035).

### Detection of externalized PIPs

Apoptosis was induced using camptothecin (10 μM), cycloheximide (200 μM), etoposide (100 μM) (ab102480, Abcam), or anti-FAS antibody (305702, 150 ng/ml, BioLegend). Ferroptosis and necroptosis were induced after treatment with selective activators, Erastin (E7781, 1 μM, Sigma–Aldrich) or L-Buthionine-sulfoximine (BSO) (B2515, 1 μM, Sigma–Aldrich) for ferroptosis and Emodin (E7881, 5 μM, Sigma–Aldrich) or Shikonin (S7576, 1 μM, Sigma–Aldrich) for necroptosis. After fixation, the cells were treated with serum-free protein blocking solution (DAKO) and incubated with anti-PIP_3_ IgM antibody (Z-P345, 1:100, Echelon Biosciences), CD14, AKT PHD, AKT-PHD/eGFP, or Annexin V. For immunofluorescence studies, tissue sections (2.5 μm) were blocked (DAKO) and incubated with anti-PI(3,4,5)P_3_ and anti-cleaved caspase 3 antibodies. After mounting, the sections were imaged with an LSM 700 laser-scanning confocal microscope (Carl Zeiss), and images were analyzed with ZEN 2010 Software (Carl Zeiss).

### Flow cytometry

Jurkat cells treated with camptothecin or FAS antibody and mouse cells were analyzed using LSRII (BD Bioscience) at Core-facility for Cell to In-vivo imaging. PI(3,4,5)P_3_ antibody, recombinant AKT PHD, Annexin V, and/or CD14 proteins were used for detection. Annexin V and AKT-PHD were labeled with FITC (53027, Pierce) or Alexa 647 (A20173, Molecular Probes). Cleaved caspase 3 (active) was assessed using Vybrant-FAM, a fluorescein-conjugated caspase 3/7 inhibitor (V35118, Molecular Probes). The data were processed using FlowJo software (Tree Star) [29].

### Statistical analysis

Unless noted otherwise, all data are presented as the mean ± standard deviation (SD). As described previously [30], group statistical comparisons were performed using unpaired Student’s *t* tests or one-way ANOVA with Tukey’s post-hoc multiple comparison test. GraphPad Prism 9.0 (GraphPad Software Inc.) was used for data analysis and graph preparation.

## Results

### CD14 binds inositol phosphates and PIPs

**Fig. 1:**
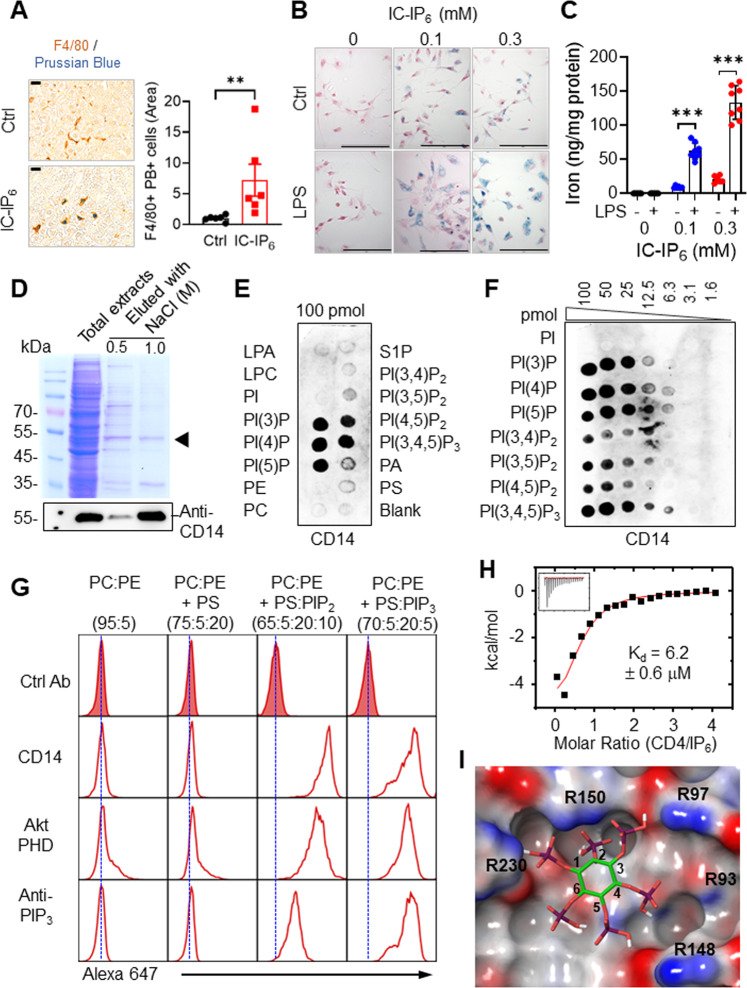
The role of CD14^+^ macrophages in IC-IP_6_ uptake and PIP recognition. Representative liver sections (left) and quantitative data (right) from mice treated with or without intravenous IC-IP_6_ (*n* = 6 per group). The liver sections were stained with Prussian blue (PB) for iron and anti-F4/80 antibodies for macrophages/Kupffer cells (scale bars, 20 μm). F4/80+ PB+ cells were quantified using ImageJ software. **B**, **C** LPS induction of IC-IP_6_ uptake by TPMs, determined by PB-stained cells (scale bars, 20 μm) (**B**), or (**C**) by iron staining with 2,4,6-tri-(2-pyridyl)-5-triazine (TPTZ) in cell lysates (*n* = 8). Each point represents the mean of three experimental replicates for each IC-IP_6_ concentration. **D** Proteins isolated from RAW264.7 cell lysates by IC-IP_6_ precipitation were eluted with NaCl, separated by SDS-PAGE, and visualized using Coomassie Brilliant Blue (black arrowhead = CD14). CD14 identity was confirmed by western blotting. **E**, **F** CD14 binding to immobilized phospholipids (100 pmol each) (**E**), and varying concentrations of phosphatidylinositol phosphates (PIP strips or PIP arrays, Echelon Biosciences) (**F**). **G** Representative flow cytometry analyses of the binding of CD14, AKT PHD, or anti-PIP_3_ antibody to the indicated phospholipids. Silica particles loaded with specific combinations of phospholipids (Echelon Bioscience) were treated with anti-PI(3,4,5)P_3_ antibody or His-tagged AKT PHD, or CD14 protein. Samples with His-tagged proteins were treated with primary anti-His antibody, and all were visualized using an Alexa-647-labeled secondary antibody. The blue dotted lines indicate the peaks of the isotype controls. **H** ITC results for IP_6_ titration into 30 μM CD14; the K_d_ value was determined by curve fitting the raw data (*n* = 2; MicroCal). **I** Modeled PI(3,4,5)P_3_-CD14 structure, generated with AutoDock PyRx and coordinates for IP_6_ (1ZY7) [50] and CD14 (1WWL) [35]; red (acidic) and blue (basic) are according to the electrostatic potential. IP_6_ potentially interacts with CD14 residues R92, R97, R150, and R230. All comparisons were performed by one-way ANOVA with Tukey’s post-hoc multiple comparison test (Fig. 1C) or unpaired student *t* test (Fig. 1A). All data are presented as mean ± standard deviation (SD) for each group. *p < 0.05, **p < 0.01, ***p < 0.001 compared to controls. See also Supplementary Fig. 1.

Intravenous administration of technetium-99m-labeled inositol hexakisphosphate (^99m^Tc-IP_6_) has been used clinically to image the reticuloendothelial system [31]. We developed a related iron-calcium derivative, iron-calcium (IC)-IP_6_ (Supplementary Fig. 1A), which is taken up by tissue macrophages [25, 26]. We visualized IC-IP_6_ uptake using Prussian Blue (PB) staining and an anti-F4/80 antibody (Fig. 1A). LPS pretreatment of thioglycolate-elicited peritoneal macrophages (TPMs) increased IC-IP_6_ uptake (Fig. 1B, C), suggesting that IC-IP_6_ uptake is mediated by an LPS-induced receptor or transporter. To identify potentially responsible proteins, we isolated IC-IP_6_-binding proteins from the lysates of LPS-stimulated RAW264.7 macrophages. Proteins separated by SDS-PAGE (Fig. 1D) were cleaved with trypsin and identified using tandem liquid chromatography-mass spectrometry. CD14 fragments were among the most abundantly identified membrane peptides (Supplementary Fig. 1B), and immunoblot analyses confirmed that CD14 was eluted from IC-IP_6_, indicating its selective binding (Fig. 1D). LPS stimulation and hypoxia both induced expression of TPM *Cd14* (Supplementary Fig. 1C, D), suggesting that elevated CD14 levels might account for the LPS-induced increase in IC-IP_6_ uptake (Fig. 1B, C). Underscoring the requirement of CD14 for IC-IP_6_ uptake, livers from IC-IP_6_-treated *Cd14*^−/−^ mice contained 9-fold less iron than those of similarly treated WT mice (Supplementary Fig. 1E).

We hypothesized that a structurally related compound to IC-IP_6_ (Supplementary Fig. 1F) is a physiologically relevant in vivo ligand for CD14. We therefore expressed full-length human CD14 in mammalian HEK293T cells and examined its binding properties toward IP_6_-related PIPs [32]. Protein-lipid overlay experiments showed selective binding of CD14 to PIPs but not to PS, PC, PE, phosphatidylinositol (PI), sphingosine 1-phosphate, lysophosphatidic acid, or lysophosphatidylcholine (Fig. 1E). CD14 bound all phosphorylated PIPs, but exhibited the greatest affinity toward PI(3,4,5)P_3_, with detectable binding at 6.3 pmol (Fig. 1F). We further analyzed the binding properties of CD14 toward various phospholipids by flow cytometry using custom-made lipid nanoparticles, which allowed lipid headgroups to be exposed for biological interactions. Consistently, flow cytometry demonstrated that CD14 bound PI(4,5)P_2_ and PI(3,4,5)P_3_ but not PC or PS (Fig. 1G). The mean fluorescence intensity (MFI) value showed that CD14 PIP-binding was highly compatible with specific AKT pleckstrin homology domains (PHDs), which play an essential role in recruiting AKT by binding to plasma membrane PI(3,4,5)P_3_ or PI(3,4)P_2_ [33, 34], using an anti-PI(3,4,5)P_3_ antibody (Supplementary Fig. 1G).

Using isothermal titration calorimetry (ITC), the gold standard for measuring binding affinity, we determined the affinities of CD14 toward soluble IP_6_ (K_d_ = 6.2 ± 1.2 μM) (Fig. 1H) and PI(3,4,5)P_3_ in liposomes (K_d_ = 5.6 ± 0.6 μM) (Supplementary Fig. 1H). Modeling studies based on the CD14 structure [35] predicted anchoring of phosphates at the 1, 3, 4, and 5 positions of IP_6_ and PI(3,4,5)P_3_ through salt bridges with side chains of R92, R148, R150, and R230 (Fig. 1I). This may explain why PI(3,4,5)P_3_ binds CD14 with the highest apparent affinity (Fig. 1F), as it is the only PIP with phosphate groups at all four positions. Substitutions of conserved arginine residues to create R92 A, R150A, or R92 A/R148A/R230A (R4A) resulted in marked decreases in CD14 binding toward all PIPs (Supplementary Fig. 1I). The high-affinity binding of PIPs (normally inwardly facing) to CD14 (normally outwardly facing) suggested that conditions exist where the two meet, i.e., either PIPs face outward or CD14 faces inward.

### Exofacial PI(3,4,5)P_3_ on apoptotic cells

**Fig. 2:**
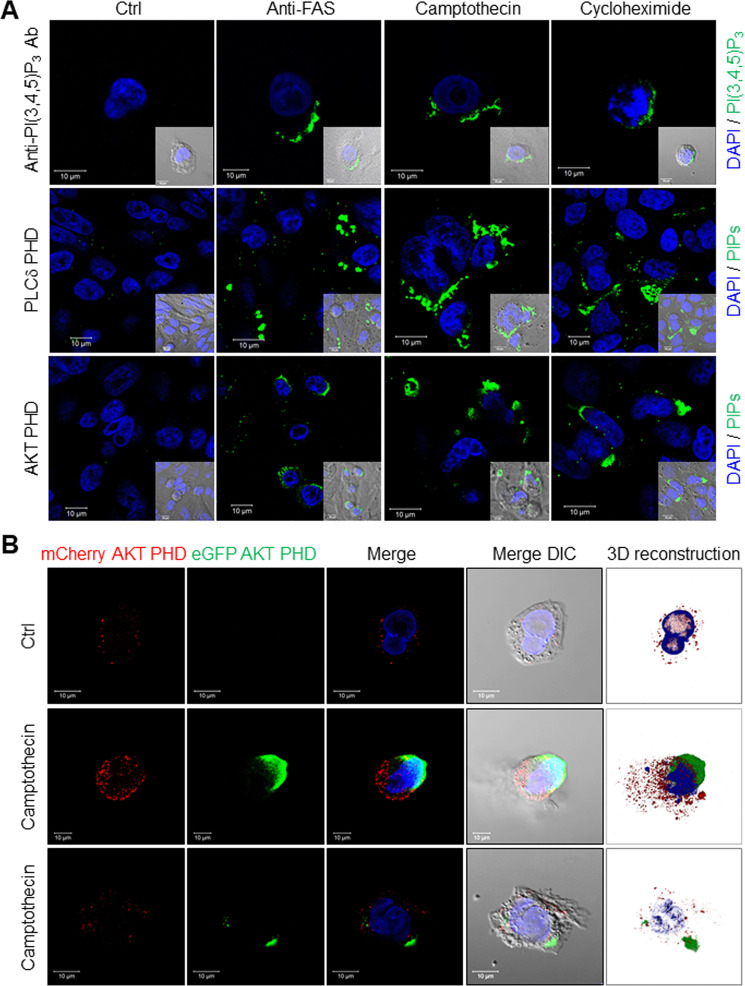
Visualizing externalized PIPs on apoptotic cells. **A** Representative images of externalized PIPs on apoptotic HeLa cells. HeLa cell apoptosis was induced by 16 h treatment with either an anti-FAS antibody (100 ng/ml), camptothecin (10 μM) or cycloheximide (100 μM). Cells were stained using an anti-PI(3,4,5)P_3_ antibody, recombinant MYC-tagged PLCδ PHD, or AKT-PHD protein. All were visualized using an FITC-labeled secondary antibody and DAPI (scale bars, 10 μm). Inserts use differential interference contrast microscopy. **B** Representative images of externalized PI(3,4)P_2_ and PI(3,4,5)P_3_ detection using a recombinant eGFP/AKT PHD fusion protein on CHO cells expressing mCherry/AKT PHD fusion protein. Apoptosis was induced in CHO cells that stably expressed the mCherry/AKT PHD fusion protein to visualize intracellular PI(3,4)P_2_ and PI(3,4,5)P_3_, while externalized PIPs were visualized using recombinant eGFP/AKT PHD fusion protein (scale bars, 10 μm). Three-dimensional reconstructed images were made using Zeiss Zen SP2 software. Camptothecin-treated cells typically displayed either a large bleb or small patches of eGFP-positive staining; examples of both are shown. Supplementary Movie 1 provides a time-lapse video. See also Supplementary Figs. 2–4.

Membrane polarity may be perturbed during apoptosis [11, 13], when normally inwardly facing PS is externalized to become an eat-me signal for macrophages [1, 2, 9, 36]. Given that CD14 may recognize apoptotic cells [21, 23, 24] but not PS [37], we asked whether PIPs were exposed outwardly during apoptosis. Under basal conditions, PI(3,4,5)P_3_-antibody binding to both Chinese hamster ovary (CHO) and HeLa cells was negative, but was readily detected after initiation of apoptosis through treatment with an anti-FAS antibody, an inhibitor of protein translation (cycloheximide), or a topoisomerase (camptothecin) (Fig. 2A and Supplementary Fig. 2A). We further tested PIP membrane polarity using the high inherent specificities of AKT and PLCδ PHDs toward PI(3,4,5)P_3_ and either PI(3,4)P_2_ or PI(4,5)P_2_, respectively [33, 34, 38]. Neither PLCδ nor AKT PHDs recognized cultured CHO cells until apoptosis had been initiated with an anti-FAS antibody, cycloheximide, or camptothecin (Fig. 2A and Supplementary Fig. 2B). The fluorescence intensity of externalized PIPs detected by anti-PIP3 antibody, PLCδ PHD, and AKT PHD, was almost 10 to 50-fold higher than the PBS control (Supplementary Fig. 2C). These results parallel those obtained with the anti-PI(3,4,5)P_3_ antibody. Additional studies using an anti-PI(4,5)P_2_ antibody confirmed its externalization by apoptotic cells, indicating that in addition to PS, several plasma membrane PIPs flip outward during apoptosis to become accessible on the cell surface (Supplementary Fig. 2D).

To further assess PIP membrane polarity and to control for the recognition of intracellular PIPs by extracellular reagents, which may penetrate the leaky plasma membranes of apoptotic cells, we stably expressed the AKT PHD/mCherry fusion protein in CHO cells that already stably expressed an insulin receptor (CHO/IR) [39].

Upon insulin stimulation, basal punctate staining with the AKTPHD/mCherry fusion protein transitioned to the inner plasma membrane (Supplementary Fig. 3A), consistent with the generation of additional PI(3,4,5)P_3_ via insulin-activated PI 3-kinase [15, 16, 34]. The exogenous AKT PHD/eGFP fusion protein was not visualized basally or after insulin stimulation (Fig. 2B). By contrast, basal intracellular PI(3,4,5)P_3_ staining with the intracellular AKT PHD/mCherry fusion protein was unchanged by camptothecin, whereas the extracellular AKT PHD/eGFP fusion protein recognized externalized PI(3,4,5)P_3_ and showed distinct focal patches on camptothecin-treated apoptotic cell surfaces in the form of either individual large blebs or multiple smaller patches (Fig. 2B, Supplementary Fig. 3B, C, and Supplementary Movie 1). These methods can clearly distinguish inwardly facing from outwardly facing PIPs, and showed that outwardly facing PI(3,4,5)P_3_ has a distinct distribution after the induction of apoptosis. In addition to apoptosis, we found that cells undergoing ferroptosis or necroptosis had externalized PI(3,4,5)P_3_ on their cell surfaces after treatment with selective activators, Erastin or L-Buthionine-sulfoximine (BSO) for ferroptosis [40, 41] and Emodin or Shikonin for necroptosis [42, 43] (Supplementary Fig. 4A–D). These results suggest that exofacial PI(3,4,5)P_3_ is a common cell death marker and not a specific event in apoptosis.

### CD14 recognizes exofacial PIPs as eat-me signals

**Fig. 3:**
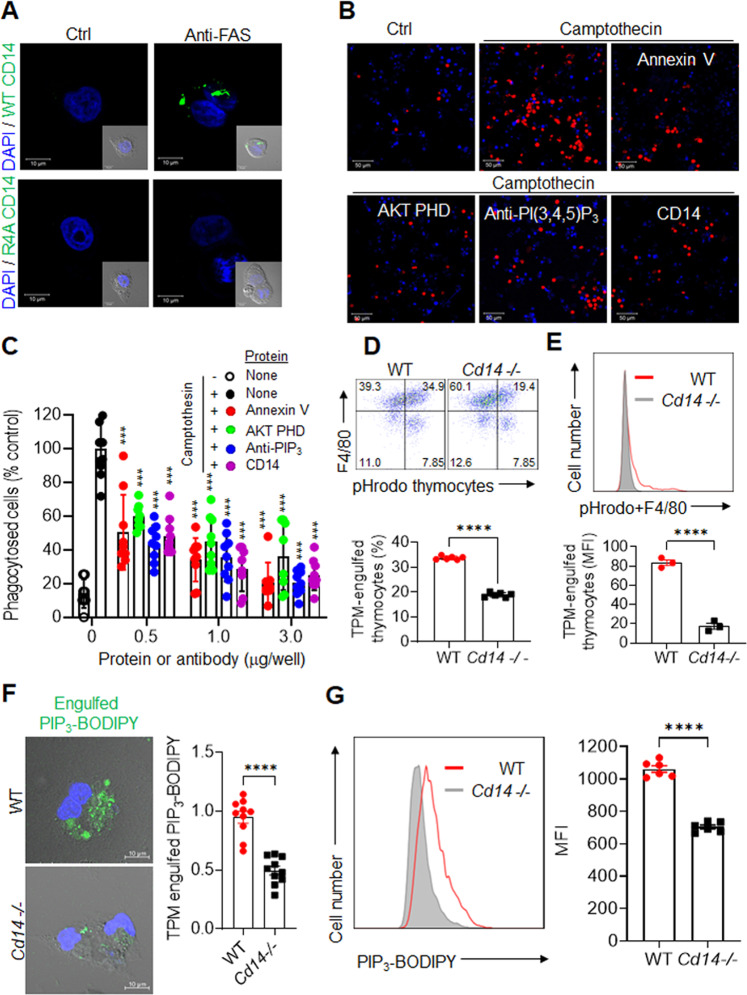
PIP externalization and its role in phagocytosis. **A** Representative images of apoptotic cell detection by recombinant WT CD14 or mutant R4A CD14 protein. After inducing apoptosis with an anti-FAS antibody (100 ng/ml), HeLa cells incubated with recombinant WT or R4A CD14 were visualized using an anti-CD14 primary antibody and an FITC-labeled secondary antibody (scale bars, 10 μm). **B** Representative images of TPM phagocytosis of camptothecin-treated, pHrodo-labeled Jurkat cells using an antibody or recombinant proteins to mask externalized PS or PIPs. TPM phagocytosis of camptothecin-treated, pHrodo-labeled Jurkat cells was inhibited by an anti-PI(3,4,5)P_3_ antibody, AKT PHD, Annexin V, and CD14 (scale bars, 50 μm). The red color represents engulfed apoptotic cells labeled with pHrodo dyes. **C** TPM phagocytosis of camptothecin-treated, pHrodo-labeled Jurkat cells was quantified in 8–10 low-power fields (mean ± SD, *n* = 5–10). **D** Representative flow cytometric plots (left) and percentages (right) of F4/80^+^ TPM phagocytosis of pHrodo-labeled thymocytes isolated from irradiated WT or *Cd14*^−/−^ mice group (*n* = 6 per group). Thymocytes from irradiated mice were labeled with pHrodo for 30 min prior to adding TPMs from WT or *Cd14*^−/−^ mice for 1 hr (mean ± SD, *n* = 6). **E** Representative flow cytometry plots (upper) and MFI values (lower) of in vivo phagocytosis of pHrodo-labeled apoptotic thymocytes by F4/80+ mouse peritoneal macrophages of WT or Cd14^−/−^ mice group (*n* = 3 per group). Thymocytes from irradiated mice were labeled with pHrodo for 30 min prior to injecting the peritoneal cavity of WT or *Cd14*^−/−^ mice for 15 min. **F** Representative fluorescent images (left) and intensities (right) of engulfed synthetic Bodipy-labeled PI(3,4,5)P_3_ in TPMs from WT or *Cd14*^*−/−*^ mice. Engulfed Bodipy-labeled PIP_3_ was visualized by confocal microscopy and quantified by fluorescence intensity using NIH ImageJ software (mean ± SD, *n* = 10) after incubating the TPMs with 1 μg of Bodipy-labeled PI(3,4,5)P_3_ per well for 30 min (scale bars, 10 μm). **G** Representative flow cytometric plots (left) and MFI values (right) of engulfed synthetic Bodipy-labeled PI(3,4,5)P_3_ in PI^-^/F4/80^+^ TPMs from WT or *Cd14*^*−/−*^ mice (mean ± SD, *n* = 6). All comparisons were performed by one-way ANOVA with Tukey’s post-hoc multiple comparison test Fig. 3A) or unpaired student *t* tests (Fig. 4D–F). All data are presented as the mean ± SD for each group. *p < 0.05, **p < 0.01, ***p < 0.001, ****p < 0.0001 compared with camptothecin-treated controls. See also Supplementary Figs. 5–7.

Recombinant CD14 also bound to the surfaces of HeLa and CHO cells after induction of apoptosis, consistent with CD14 recognition of externalized PIPs (Fig. 3A and Supplementary Fig. 5A, B). By contrast, R4A CD14 did not bind PIPs either in biochemical assays or on apoptotic cells (Fig. 3A). Furthermore, the fluorescence intensity of externalized PIPs by WT CD14 and R4A CD14 mutant protein was almost 30-fold higher than those of the PBS control (Supplementary Fig. 5C). To further assess externalized PIP dependence for macrophage phagocytosis, we performed an in vitro phagocytosis assay using pHrodo-labeled apoptotic Jurkat cells [12]. LPS-stimulated TPMs rarely phagocytize healthy Jurkat cells, but treatment with camptothecin caused 10- to 20-fold increases in the phagocytosis of pHrodo-labeled Jurkat cells (Fig. 3B). The Jurkat-cell engulfment was inhibited in a dose-dependent manner following pre-incubation with a PIP-specific antibody (anti-PI(3,4,5)P_3_), AKT PHD, CD14, or Annexin V (Fig. 3C). Annexin V, an established and widely used reagent for identifying apoptotic cells, binds to externalized PS [5, 36, 44, 45]. Furthermore, engulfment of apoptotic HeLa or CHO cells by phorbol myristate acetate (PMA)/LPS-stimulated human THP1 cells (Supplementary Fig. 6A–D) and engulfment of apoptotic thymocytes by murine RAW264.7 macrophages (Supplementary Fig. 6E, F) were also inhibited following pre-incubation with AKT PHD, CD14, or annexin V. The results show that the masking PIPs or PS on the apoptotic cell surface inhibits phagocytosis by macrophages from the same species as well as different species. By contrast, TPM phagocytosis of labeled bacteria (*Escherichia coli*) or synthetic beads was not inhibited after incubation with AKT PHD, Annexin V, or CD14 (Supplementary Fig. 7a), suggesting that PS- or PIP-mediated recognition and engulfment were selective for apoptotic cells and are not involved in either bacterial or latex-bead phagocytosis.

The potential role of CD14 in cellular phagocytosis was further assessed using TPMs isolated from *Cd14*^−/−^ mice. *Cd14*^−/−^ TPMs engulfed fewer pHrodo-labeled apoptotic Jurkat cells than WT TPMs (Fig. 3D), despite upregulation of CD36 and other putative eat-me receptors on *Cd14*^−/−^ TPMs [1, 3] (Supplementary Fig. 7B). To confirm the biological significance of these in vitro results, we performed in vivo phagocytosis assays by injecting pHrodo-labeled apoptotic thymocytes (8 × 10^6^ cells per mouse) from irradiated C57BL/6 J mice into the peritoneal cavity of either WT or Cd14^−/−^ mice. Consistent with in vitro phagocytosis results, in vivo engulfment of pHrodo-labeled apoptotic thymocytes in *Cd14*^−/−^ peritoneal macrophages was dramatically less than in WT peritoneal macrophages (Fig. 3E), indicating that loss of CD14 lead to impaired apoptotic thymocyte clearance by phagocytes. To further establish that CD14 recognizes externalized PIP_3_, we incubated WT and *Cd14*^−/−^ TPMs with synthetic Bodipy-labeled PI(3,4,5)P_3_ (PIP_3_-Bodipy) (Supplementary Fig. 7C). Confocal immunofluorescence microscopy and flow cytometry showed that the engulfment of PIP_3_-Bodipy was markedly decreased in *Cd14*^−/−^ TPMs relative to WT TPMs (Fig. 3E, F). These findings indicate that externalized PIPs serve as eat-me signals for apoptotic cells, CD14 is a phagocyte eat-me signal receptor for externalized PIPs, and externalized PIPs on apoptotic cells and CD14 receptor on macrophages are sufficient for the recognition and engulfment of apoptotic cells.

### Kinetics of exofacial PIPs

**Fig. 4:**
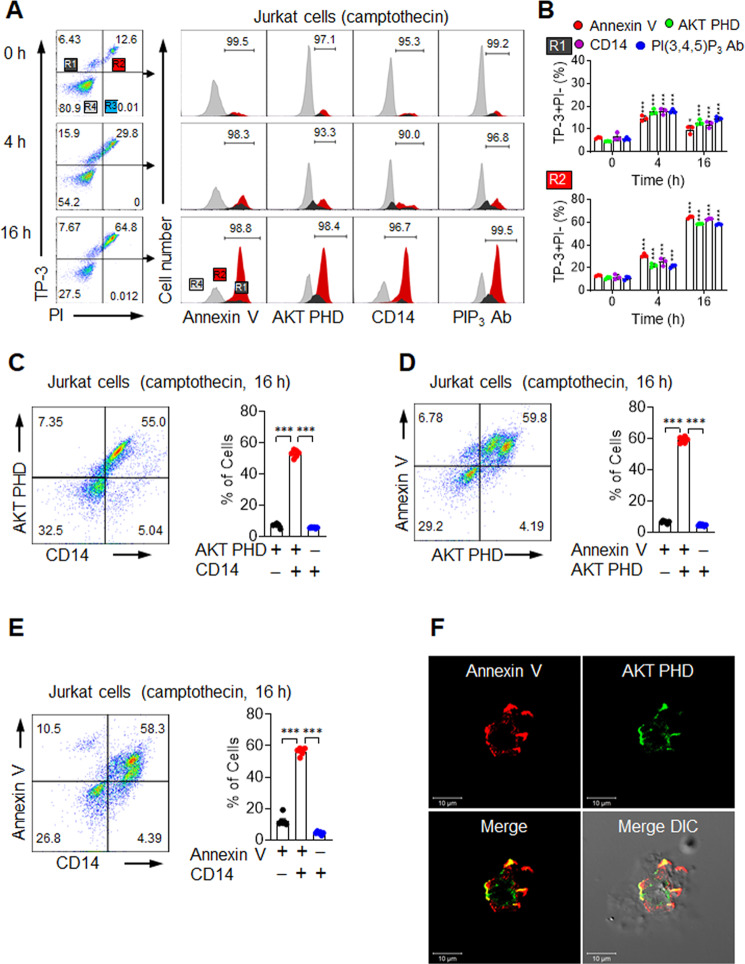
In vitro induction of exofacial PIPs. **A**, **B** Flow cytometric analyses of Jurkat cells treated 0–16 h with 10 μM camptothecin. **A** Representative flow cytometric plots (left) of TO-PRO-3 (TP-3) and propidium iodide (PI)-stained cells showing R1 (TP-3^+^PI^−^), R2 (TP-3^+^PI^+^), R3 (TP-3^−^PI^+^), and R4 (TP-3^−^PI^−^) subsets. Histograms (right) show the binding levels of Annexin V, AKT PHD, CD14, or PIP_3_ antibody in R1 (dark gray), R2 (red), and R4 (light grey) subsets. **B** Percentage of Annexin V, AKT PHD, CD14, and PI(3,4,5)P_3_ in R1 (upper low) and R2 (lower low) cells. **C**, **D** Flow cytometric analyses of Jurkat cells treated 16 h with 10 μM camptothecin. **C** Representative flow cytometric plots (left) and binding percentages (right) of an AKT PHD and CD14 combination. **D** Representative flow cytometric plots (left) and binding percentages (right) of an Annexin V and AKT PHD combination. **E** Representative flow cytometric plots (left) and binding percentages (right) of an Annexin V and CD14 combination. **F** CHO cells treated 6 h with 10 μM camptothecin and incubated with Annexin V and AKT PHD were detected with primary antibodies and Texas Red- or FITC-labeled secondary antibodies, respectively. See also Supplementary Fig. 8 and Supplementary Movie 2. All comparisons were performed by one-way ANOVA with Tukey’s post-hoc multiple comparison test. All data are presented as the mean ± SD for each group (*n* = 6 per group unless noted otherwise). *p < 0.05, **p < 0.01, ***p < 0.001 compared to double-positive subsets.

We further used flow cytometry and the nucleic acid-binding dye TO-PRO-3 (TP-3) to assess the kinetics and specificity of these interactions and to distinguish early apoptotic from late-apoptotic and necrotic cells [46]. TP-3^+^PI^−^ (R1) and TP-3^+^PI^+^ (R2) staining can be used to distinguish between early and late apoptosis, and both are distinct from TP-3^−^PI^+^ (R3) necrosis. Early apoptotic R1 cells showed nearly identical signals for Annexin V, AKT PHD, CD14, and anti-PIP_3_ antibody (Fig. 4A, B). Similarly, late-apoptotic R2 cells also showed similar results for Annexin V, AKT PHD, CD14, and anti-PIP_3_ antibody, regardless of camptothecin treatment time (Fig. 4A, B). We investigated this result further using a double-staining approach. The majority of cells labeled with mixtures of AKT-PHD and CD14 were doubly positive (Fig. 4C), consistent with both reagents recognizing externalized PIP signals on apoptotic cells. Similarly, most cells were doubly positive when stained with any pair of Annexin V, AKT PHD, and CD14 (Fig. 4D, E). Confocal immunofluorescence microscopy confirmed that both Annexin V and AKT PHD bound to apoptotic cells with similar patchy distributions (Fig. 4F, Supplementary Fig. 8A, B, and Supplementary Movie 2). Given the similar externalization kinetics and cell surface distributions of PS and PIPs, both may serve as eat-me signals for the clearance of apoptotic cells.

### In vivo exposure of PIP eat-me signals

**Fig. 5:**
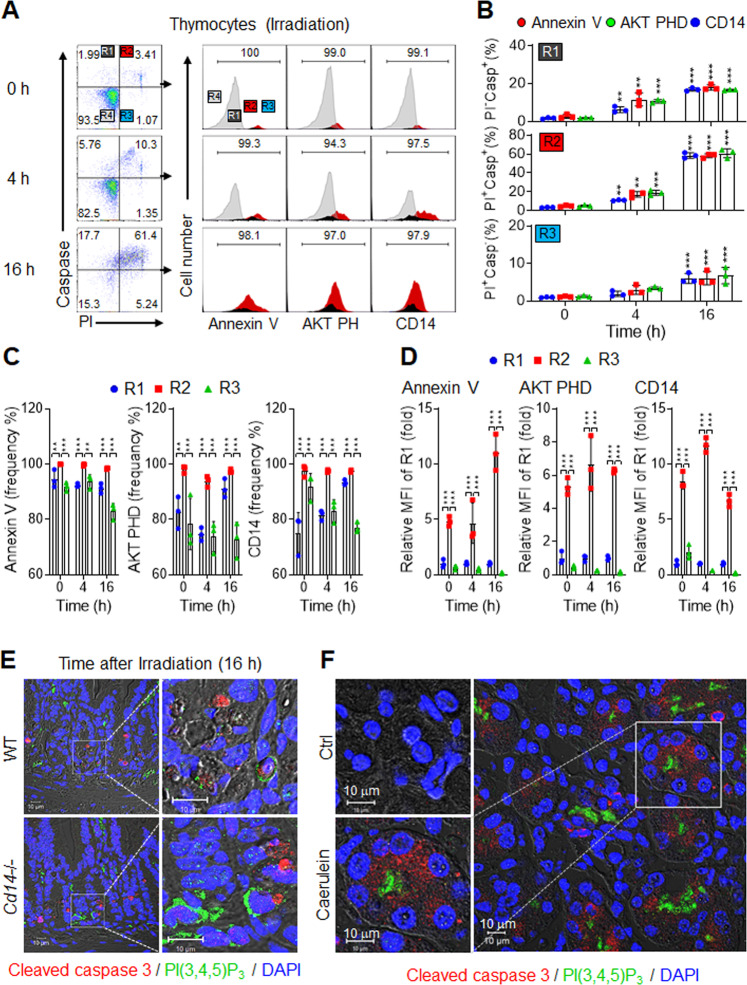
In vivo induction of exofacial PIPs. **A**–**D** Flow cytometric analyses of thymocytes from mice 0–16 h after irradiation (10 Gy). **A** Representative flow cytometric plots (left) of propidium iodide (PI) and cells stained for caspase 3/7 covalent suicide inhibitor (Casp) showing the R1 (PI^−^Casp^+^), R2 (PI^+^Casp^+^), R3 (PI^+^Casp^−^), and R4 (PI^−^Casp^−^) subsets. Histograms (right) show the binding levels of Annexin V, AKT PHD, or CD14 in R1 (dark grey), R2 (red), R3 (light blue), and R4 (light grey) subsets (*n* = 3). **B** Percentages of Annexin V, AKT PHD and CD14 binding for R1 (upper row), R2 (middle row) and R3 (lower row) cells. **C** Frequencies of Annexin V, AKT PHD, and CD14 in R1, R2, and R3 thymocytes. **D** The relative MFI values in R1, R2, and R3 cells for AKT PHD, Annexin V, and CD14 binding. The MFI values were normalized to those of R1 at each time point. **E** Representative images of jejunum sections from irradiated WT and *Cd14*^−/−^ mice were stained for cleaved caspase 3 (red), PI(3,4,5)P_3_ (green), and DAPI (blue). **F** Representative images of pancreatic sections from mice treated with caerulein [47] stained with DAPI (blue), cleaved caspase 3 (red), and PI(3,4,5)P_3_ (green). Scale bars, 10 μm. See also Supplementary Fig. 9. All comparisons were performed by one-way ANOVA with Tukey’s post-hoc multiple comparison test. All data are presented as mean ± SD for each group (*n* = 3 per group). *p < 0.05, **p < 0.01, ***p < 0.001 compared to R1 subsets.

To determine whether PIP externalization similarly characterizes apoptotic cells in vivo, we irradiated C57Bl/6 J mice to broadly induce apoptosis in susceptible tissues. Propidium iodide and a fluorescent probe for cleaved, activated caspase 3 (Casp^+^; FAM-FLICA, Molecular Probes) were used to relate PIP externalization to the kinetics of apoptosis. Thymocyte apoptosis was readily apparent after irradiation, as numbers of early apoptotic R1 thymocytes and late-apoptotic R2 thymocytes continually increased over time after irradiation (Fig. 5A, B). Consistent with the in vitro results, 74–94% of early apoptotic R1 thymocytes were AKT PHD^+^, CD14^+^, or Annexin V^+^, whereas 94–100% of late-apoptotic R2 thymocytes were AKT PHD^+^, CD14^+^, or Annexin V^+^. These findings indicate that cells entering apoptosis often became Casp^+^ prior to displaying exofacial PS or PIPs, whereas nearly all late-apoptotic R2 thymocytes displayed externalized PS and PIPs (Fig. 5C). R3 thymocytes may be necrotic rather than apoptotic [5], but they too externalize PS and PIPs. However, the MFI values were markedly lower in R3 compared to R2 thymocytes (Fig. 5D), suggesting that externalized PS and PIP abundance were substantially lower on R3 than R2 thymocytes. Similar to PS exposure, these findings suggest that neither PS nor PIPs externalize early, but instead, both may serve as a marker of mid-to-late apoptosis in vivo and in vitro.

The rapidly dividing cells in intestinal crypts are highly radiation-sensitive. In WT mice, the numbers of Casp^+^ apoptotic cells assessed through immunohistochemistry (IHC) in the jejunum increased by 10- and 20-fold 4 h and 16 h post-irradiation, respectively (Supplementary Fig. 9A, B). We also analyzed *Cd14*^−/−^ mice under similar conditions. Prior to irradiation, the number of Casp^+^ apoptotic cells was 5-fold more significant in *Cd14*^−/−^ mice than in WT mice, consistent with CD14 playing a role in the recognition of apoptotic cells [21, 23, 24]. As in WT mice, Casp^+^ cells in Cd14^−/−^ mice increased after irradiation by 5.6- and 6.6-fold at 4 h and 16 h, respectively. Because the baseline number of apoptotic cells was higher in *Cd14*^−/−^ mice, the fold-change after irradiation was smaller for *Cd14*^−/−^ mice than for WT mice, but the absolute number of apoptotic cells was always higher in *Cd14*^−/−^ mice than in WT mice (Supplementary Fig. 9A, B). Clearance mechanisms appeared to become saturated in *Cd14*^−/−^ mice, but not in WT mice. Next, we asked if PI(3,4,5)P_3_ was externalized on apoptotic cells of intestinal crypts. Casp^+^ cells were stained using anti-PI(3,4,5)P_3_ antibody, and the results supported exofacial PI(3,4,5)P_3_ as a marker of apoptosis (Fig. 5E, Supplementary Fig. 9C).

As an alternative to irradiation, we asked whether PIP externalization accompanied treatment with caerulein, an exocrine secretagogue peptide that causes acute pancreatitis in rodents due to acinar cell apoptosis [47] and necroptosis [48]. Pancreas sections showed abundant Casp^+^ staining within the central exocrine acini after caerulein treatment (Fig. 5F, Supplementary Fig. 9D, E). Staining of PI(3,4,5)P_3_ was readily apparent on the surfaces of the same cells. Therefore, in both of these in vivo settings, Casp^+^ cells were also positive for exofacial PI(3,4,5)P_3_, consistent with both the in vitro results and with PIPs functioning as eat-me signals for the clearance of apoptotic cells by CD14-expressing phagocytes.

## Discussion

PIPs are mainly concentrated on the cytosolic surfaces of membranes [49]. Several PI kinases phosphorylate the 3, 4, and 5 hydroxyl positions of the inositol head group to yield seven distinct PIPs, each of which is uniquely distributed on cytosolic surfaces and subcellular organelles [14]. The PIPs dynamically participate in various distinct intracellular processes [16, 33], including lipid signaling, cell signaling, cytoskeletal reorganization, and membrane trafficking. Thus, PIPs orchestrate intracellular signaling effects to act as docking lipids to recruit cytosolic proteins or cytosolic domains of membrane proteins [14, 16, 33]. In contrast to the traditional roles of PIPs as pleiotropic regulators of intracellular signaling, the involvement of exofacial PIPs in apoptotic cell death and the physiological functions of exofacial PIPs have not been highlighted.

Using affinity-based proteomics, we identified that CD14 binding is required for the LPS-induced uptake of IC-IP_6_ by both tissue-resident and peritoneal macrophages. We further showed that CD14 binds to structurally related PIPs, including PI(3,4,5)P_3_. Given that apoptotic cells were previously shown to accumulate in mice lacking CD14 [21] and that CD14 mediates the uptake and metabolism of extracellular PIPs [22], we hypothesized that inwardly facing PIPs flip within the plasma membrane to expose their headgroups, similar to what is thought to occur for PS. Readily available and highly specific reagents for the identification of PI(3,4,5)P_3_, PI(3,4)P_2_, and PI(4,5)P_2_ confirmed this hypothesis, including PI(3,4,5)P_3_ and PI(4,5)P_2_ antibodies and PLCδ and AKT PHDs [33, 38]. These reagents enabled clear visualization of small patches of exposed PIPs on some cells and larger membrane blebs on others, which was consistent with known apoptotic cell morphologies. The same reagents also showed that PIPs were exposed on apoptotic cell surfaces in vivo, especially after induction of apoptosis with irradiation or caerulein although a few intracellular PIPs can be exposed during tissue section process.

The reagents used to visualize externalized PIPs, including the antibody to PI(3,4,5)P_3_ and AKT PHD also blocked phagocytosis of apoptotic Jurkat cells by peritoneal macrophages. These findings suggest that externalized PIPs are sufficient for phagocytic engulfment, although relative roles for PS, PIPs, and possibly other eat-me signals remain to be established. Recombinant CD14 blocked phagocyte engulfment, consistent with its role as an eat-me signal receptor for endogenous exofacial PIPs. Specifically, we confirmed that *Cd14*^−/−^ macrophages exhibited decreased uptake of synthetic Bodipy-labeled PIP_3_ compared to WT macrophages, suggesting that CD14^+^ macrophages are responsible for the recognition of externalized PIPs on apoptotic cell surfaces. These results are supported by the impaired ability of *Cd14*^−/−^ macrophages to engulf apoptotic cells. Apoptotic cells have been shown to accumulate in *Cd14*^−/−^ mice, but not in great numbers, consistent with the idea that additional mechanisms exist for apoptotic cell clearance [1, 3].

Of note, the apoptosis rates and the spatiotemporal distributions of putative eat-me signal(s) were virtually identical for the PIP-specific antibodies and AKT PHD, as well as for CD14 and Annexin V. These reagents clearly identified the same apoptotic cells, regardless of whether PS or PIPs were recognized. Undoubtedly, the cellular machinery for maintaining plasma membrane phospholipid asymmetry affects both PS and PIPs, and the breakdown of this energy-dependent process during apoptosis causes a loss of membrane polarity of both PS and PIPs. Consistent with this possibility, we found that, like PS, PIP exposure occurs after caspase activation and, therefore, late in the apoptotic process.

PS has been intensively studied as an eat-me signal [9]. By contrast, previous studies have neither identified PIPs as eat-me signals nor identified CD14 as a PIP receptor [21]. However, CD14 functions as a co-receptor along with TLR4 and MD-2 for the recognition and endocytosis of bacterial LPS, suggesting that CD14 may also interact with components of this pathway to recognize apoptotic cells. Consistently, *Cd14*^−/−^ macrophages similarly failed to engulf apoptotic thymocytes. Additional experiments are required to determine whether TLR4 is involved in the clearance of apoptotic cells. Notably, reagents, such as anti-PI(3,4,5)P_3_ antibodies, AKT PHD, and recombinant CD14, which reduced the engulfment of apoptotic cells by TPM, did not block the engulfment of bacteria or latex beads. These reagents do not disrupt phagocytosis in general but instead are selective for PIP-mediated mechanisms. We conclude that apoptotic cells externalize PIP eat-me signals that are recognized and cleared by CD14^+^ phagocytes.

## Supplementary information

Pre-Authorship form

Supplementary Fig.1

Supplementary Fig. 2

Supplementary Fig.3

Supplementary Fig.4

Supplementary Fig.5

Supplementary Fig.6

Supplementary Fig.7

Supplementary Fig. 8

Supplementary Fig.9

Reporting Checklist

Supplementary information

Representative video of externalized PI(3,4)P2 and PI(3,4,5)P3 detection using a recombinant eGFP/AKT PHD fusion protein on CHO cells expressing mCherry/AKT PHD fusion protein.

Supplementary movie 2. Representative video of Annexin V and AKT PHD binding of apoptotic cells.

## Data Availability

The detailed procedures of Methods, five Figures, two Supplementary Movies, and nine Supplementary Figures are attached.
