# Supplementary material for: Externalized phosphatidylinositides on apoptotic cells are eat-me signals recognized by CD14
Source: Cell Death Differ. 2022 Jan 11;29(7):1423–32. doi: 10.1038/s41418-022-00931-2 (PMC9287416; doi:10.1038/s41418-022-00931-2)
Supplement: Supplementary file 1 — Pre-Authorship form [file 41418_2022_931_MOESM1_ESM.pdf]

SPRINGER NATURE

Change of authorship request form - Journals (pre-acceptance)

Important information. Please read.

- This form should be used by authors to request any change in authorship (adding/deleting authors) including changes in corresponding authors. This form should not be used for name changes. Please fully complete all sections. Use black ink and block capitals and provide each author's full name with the given name first followed by the family name.

- By signing this declaration, all authors guarantee that the order of the authors are in accordance with their scientific contribution, if applicable as different conventions apply per discipline, and that only authors have been added who made a meaningful contribution to the work.

- Please note, in author collaborations where there is formal agreement for representing the collaboration, it is sufficient for the representative or legal guarantor (usually the corresponding author) to complete and sign the Authorship Change Form on behalf of all authors, **next to the added/removed author(s)**. **(Complete Section 3, followed by Section 6.)**
  In author collaborations where there is no formal agreement for representing the collaboration and **there are more than 10 authors**, one may sign for all, provided the signer appends correspondence that attests that each of the authors have agreed to the change **and the added/removed authors sign the form**. **(Complete Section 3, followed by Section 6.)**

- Please note, we cannot investigate or mediate any authorship disputes. If you are unable to obtain agreement from all authors (including those who you wish to be removed) you must refer the matter to your institution(s) for investigation. Please inform us if you need to do this.

- If you are not able to return a fully completed form within **30 days** of the date that it was sent to the author requesting the change, we may have to withdraw your manuscript. We cannot publish manuscripts where authorship has not been agreed by all authors (including those who have been removed).

- Incomplete forms will be rejected.

- Please return/upload this form, fully completed, to the Journals Editorial Office. The Journal and/or Publisher will consider the information you have provided to decide whether to approve the proposed change in authorship. We may decide to contact your institution for more information or undertake a further investigation, if appropriate, before making a final decision.

SPRINGER NATURE Springer Nature is one of the world's leading global research, educational and professional publishers, created in May 2015 through the combination of Nature Publishing Group, Palgrave Macmillan, Macmillan Education and Springer Science+Business Media.

SPRINGER NATURE

Change of authorship request form - Journals (pre-acceptance)

Section 1: Please provide the current title of manuscript

Manuscript ID no.: CDD-21-1573

Title: **Externalized phosphatidylinositides on apoptotic cells are eat-me signals recognized by CD14**

Section 2: Please provide the previous authorship, in the order shown on the manuscript before the changes were introduced. Please indicate the corresponding author by adding (CA) behind the name.

	First name(s)	Family name	ORCID or SCOPUS id, if available
1st author	Ok-Hee	Kim	0000-0003-3801-9463
2nd author	Geun-Hyung	Kang	0000-0001-6803-8396
3rd author	June	Hur	
4th author	Jinwook	Lee	0000-0001-6013-0526
5th author	YunJae	Jung	0000-0002-1574-696X
6th author	Hookeun	Lee	0000-0002-0696-8421
7th author	Seung-Yong	Seo	0002-2672-4707
8th author	Dae Ho	Lee	0000-0002-8832-3052
9th author	Cheol Soon	Lee	0000-0002-7191-6791
10th author	In-Kyu	Lee	0000-0002-2261-7269
11th author	Susan	Bonner-Weir	0000-0003-4682-0656
12th author	Jongsoon	Lee	0000-0002-0891-5059
13th author	Young Joo	Park	0000-0002-3671-6364
14th author	Hyeonjin	Kim	0000-0003-2693-9983
15th author	Steven E.	Shoelson (CA)	0000-0003-2609-7472
16th author	Byung-Chul	Oh (CA)	0000-0002-4277-7083
17th author			

Please use an additional sheet if there are more than 10 author

---
SPRINGER NATURE Springer Nature is one of the world's leading global research, educational and professional publishers, created in May 2015 through the combination of Nature Publishing Group, Palgrave Macmillan, Macmillan Education and Springer Science+Business Media.

SPRINGER NATURE

Change of authorship request form - Journals (pre-acceptance)

Section 3: Please provide a justification for change. Please use this section to explain your reasons for changing the authorship of your manuscript, e.g. what necessitated the change in authorship? Please refer to the (journal) policy pages for more information about authorship. Please explain why omitted authors were not originally included and/or why authors were removed on the submitted manuscriptSection 4: Proposed new authorship. Please provide your new authorship list in the order you would like it to appear on the manuscript. Please indicate the corresponding author byadding (CA) behind the name. If the Corresponding Author has changed, please indicate the reason under section 3.

During revision experiments, we performed several experiments such as confocal microscopy, flow cytometry, and in vivo phagocytosis. Professor In-Sun Hong helps us with *in vivo* phagocytosis experiments for experimental optimizations and data analysis. Therefore, we invited professor Hong as 6th coauthor in the manuscript. All the authors agreed with professor Hong's authorship. We also add professor In-Sun Hong's ORCID ID as follow: 0000-0002-3267-0127

	First name(s)	Family name (this name will appear in full on the final publication and will be searchable in various abstract and indexing databases)	Affiliated institute	E-mail address
1st author	Ok-Hee	Kim	Gachon University College of Medicine	ohkim323@gmail.com
2nd author	Geun-Hyung	Kang	Gachon University College of Medicine	rkdrmsgud@nate.com
3rd author	June	Hur	Gachon University College of Medicine	nascicell@naver.com
4th author	Jinwook	Lee	Gachon University College of Medicine	jinoogi87@naver.com
5th author	YunJae	Jung	Gachon University College of Medicine	yjjung7835@gmail.com
6th author	In-Sun	Hong	Gachon University College of Medicine	hongstem@gachon.ac.kr
7th author	Hookeun	Lee	Gachon University College of Medicine	hklee@gachon.ac.kr
8th author	Seung-Yong	Seo	Gachon University College of Medicine	syseo@gachon.ac.kr
9th author	Dae Ho	Lee	Gachon University Gil Medical Center	drhormone@naver.com
10th author	Cheol Soon	Lee	Korea University Anam Hospital	xtal21@gmail.com
11th author	In-Kyu	Lee	Kyungpook National University	leei@knu.ac.kr
12th author	Susan	Bonner-Weir	Joslin Diabetes Center	Susan.Bonner-Weir@joslin.harvard.edu
13th author	Jongsoon	Lee	SIMS	jongsoon.lee@sch.ac.kr
14th author	Young Joo	Park	Seoul National University Hosptial	yjparkmd@snu.ac.kr
15th author	Hyeonjin	Kim	Seoul National University	rachmaninov_forever@yahoo.com
16th author	Steven E.	Shoelson (CA)	Joslin Diabetes Center	steven.shoelson@joslin.harvard.edu
17th author	Byung-Chul	Oh (CA)	Gachon University College of Medicine	bcoh@gachon.ac.kr

Please use an additional sheet if there are more than 10 authors.

SPRINGER NATURE Springer Nature is one of the world's leading global research, educational and professional publishers, created in May 2015 through the combination of Nature Publishing Group, Palgrave Macmillan, Macmillan Education and Springer Science+Business Media.

SPRINGER NATURE

Change of authorship request form - Journals (pre-acceptance)

Section 5: Author contribution, Acknowledgement and Disclosures. Please use this section to provide a new disclosure statement and, if appropriate, acknowledge any contributors who have been removed as authors and ensure you state what contribution any new authors made (if applicable per the journal or book (series) policy). Please ensure these are updated in your manuscript - after approval of the change(s) - as our production department will not transfer the information in this form to your manuscript.

New acknowledgements:

New Disclosures (financial and non-financial interests, funding):

New Author Contributions statement (if applicable per the journal policy):

In-Sun Hong; designed and performed experiments and analyzed data.

State 'Not applicable' if there are no new authors.

SPRINGER NATURE Springer Nature is one of the world's leading global research, educational and professional publishers, created in May 2015 through the combination of Nature Publishing Group, Palgrave Macmillan, Macmillan Education and Springer Science+Business Media.

SPRINGER NATURE Change of authorship request form - Journals (pre-acceptance)

Section 6: Declaration of agreement. *All* authors, unchanged, new and removed *must* sign this declaration.
(NB: Please print the form, (docu)-sign and return/upload a scanned copy. Please note that signatures that have been inserted as an image file are acceptable as long as it is handwritten. Typed names in the signature box are unacceptable.) *** Please delete as appropriate. Delete all of the bold if you were on the original authorship list and are remaining as an author.**

	First name	Family name		Signature	Date
1st author	Ok-Hee	Kim	I agree to the proposed new authorship shown in section 4 /**and the addition/removal*of my name to the authorship list** /and the proposed change in corresponding author	[illegible]	Nov 3,2021
2nd author	Geun-Hyung	Kang	I agree to the proposed new authorship shown in section 4 /**and the addition/removal*of my name to the authorship list** /and the proposed change in corresponding author	[illegible]	Nov 3,2021
3rd author	June	Hur	I agree to the proposed new authorship shown in section 4 /**and the addition/removal*of my name to the authorship list** /and the proposed change in corresponding author	[illegible]	Nov 3,2021
4th authors	Jinwook	Lee	I agree to the proposed new authorship shown in section 4 /**and the addition/removal*of my name to the authorship list** /and the proposed change in corresponding author	Jinwook Lee	Nov 3,2021
5th author	YunJae	Jung	I agree to the proposed new authorship shown in section 4 /**and the addition/removal*of my name to the authorship list** /and the proposed change in corresponding author	yunjae	Nov 3,2021
6th author	In-Sun	Hong	I agree to the proposed new authorship shown in section 4 /**and the addition/removal*of my name to the authorship list** /and the proposed change in corresponding author	[illegible]	Nov 3,2021
7th author	Hookeun	Lee	I agree to the proposed new authorship shown in section 4 /**and the addition/removal*of my name to the authorship list** /and the proposed change in corresponding author	[illegible]	Nov 3,2021

SPRINGER NATURE Springer Nature is one of the world's leading global research, educational and professional publishers, created in May 2015 through the combination of Nature Publishing Group, Palgrave Macmillan, Macmillan Education and Springer Science+Business Media.

Change of authorship request form - Journals (pre-acceptance)

8th author	Seung-Yong	Seo	I agree to the proposed new authorship shown in section 4 /**and the addition/removal*of my name to the authorship list** /and the proposed change in corresponding author		Nov 3,2021
9th author	Dae Ho	Lee	I agree to the proposed new authorship shown in section 4 /**and the addition/removal*of my name to the authorship list** /and the proposed change in corresponding author		Nov 3,2021
10th author	Cheol Soon	Lee	I agree to the proposed new authorship shown in section 4 /**and the addition/removal*of my name to the authorship list** /and the proposed change in corresponding author		Nov 3,2021
11th author	In-Kyu	Lee	I agree to the proposed new authorship shown in section 4 /**and the addition/removal*of my name to the authorship list** /and the proposed change in corresponding author		Nov 3,2021
12th author	Susan	Bonner-Weir	I agree to the proposed new authorship shown in section 4 /**and the addition/removal*of my name to the authorship list** /and the proposed change in corresponding author		Nov 2,2021
13th author	Jongsoon	Lee	I agree to the proposed new authorship shown in section 4 /**and the addition/removal*of my name to the authorship list** /and the proposed change in corresponding author		Nov 3,2021
4th author	Young Joo	Park	I agree to the proposed new authorship shown in section 4 /**and the addition/removal*of my name to the authorship list** /and the proposed change in corresponding author		Nov 3,2021
15th author	Hyeonjin	Kim	I agree to the proposed new authorship shown in section 4 /**and the addition/removal*of my name to the authorship list** /and the proposed change in corresponding author		Nov 3,2021

SPRINGER NATURE Springer Nature is one of the world's leading global research, educational and professional publishers, created in May 2015 through the combination of Nature Publishing Group, Palgrave Macmillan, Macmillan Education and Springer Science+Business Media.

SPRINGER NATURE

Change of authorship request form - Journals (pre-acceptance)

16th author	Steven E.	Shoelson (CA)	I agree to the proposed new authorship shown in section 4 /**and the addition/removal*of my name to the authorship list** /and the proposed change in corresponding author	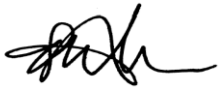	Nov 2,2021
17th author	Byung-Chul	Oh (CA)	I agree to the proposed new authorship shown in section 4 /**and the addition/removal*of my name to the authorship list** /and the proposed change in corresponding author	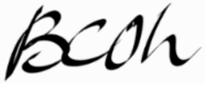	Nov 3,2021

SPRINGER NATURE Springer Nature is one of the world's leading global research, educational and professional publishers, created in May 2015 through the combination of Nature Publishing Group, Palgrave Macmillan, Macmillan Education and Springer Science+Business Media.

SPRINGER NATURE

Change of authorship request form - Journals (pre-acceptance)

Please use an additional sheet if there are more than 10 authors.

In case of author collaborations with formal agreement:

	Name of consortium/consortia	First name	Family name		Signature	Date
Representative/legal guarantor				I agree to the proposed new authorship shown in section 4 /**and the addition/removal*of my name to the authorship list** /and the proposed change in corresponding author		

Both added/removed authors should complete the information in the first table under Section 6.

---- End of form ----

SPRINGER NATURE Springer Nature is one of the world's leading global research, educational and professional publishers, created in May 2015 through the combination of Nature Publishing Group, Palgrave Macmillan, Macmillan Education and Springer Science+Business Media.
