# Supplementary figures and images for: Externalized phosphatidylinositides on apoptotic cells are eat-me signals recognized by CD14
Source: Cell Death Differ. 2022 Jan 11;29(7):1423–32. doi: 10.1038/s41418-022-00931-2 (PMC9287416; doi:10.1038/s41418-022-00931-2)

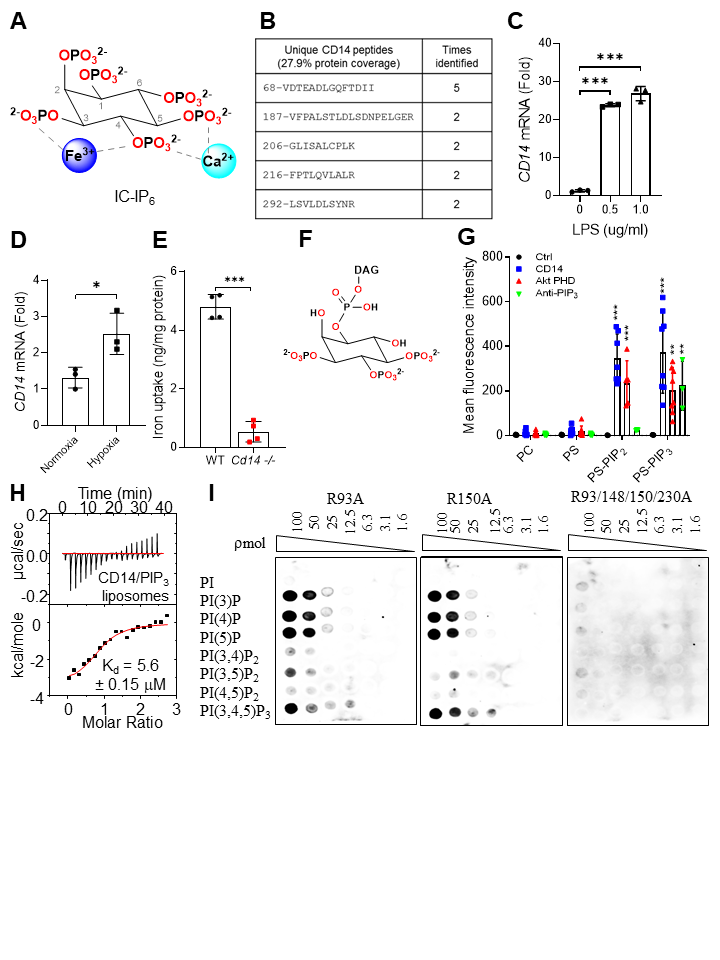

Supplement: Supplementary file 2 — Supplementary Fig.1 [file 41418_2022_931_MOESM2_ESM.tif]

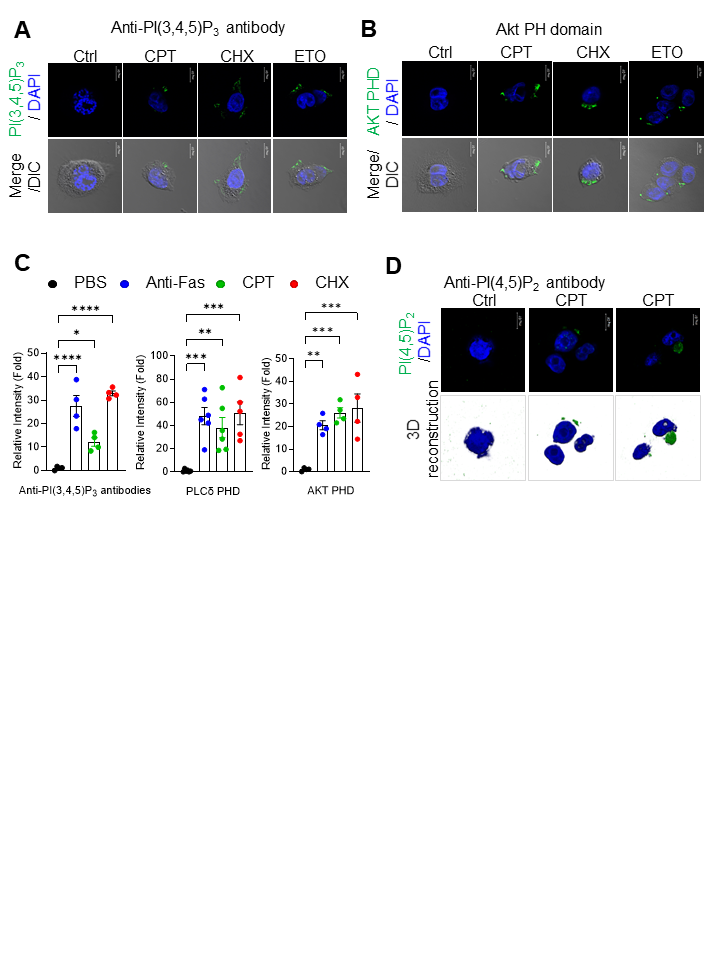

Supplement: Supplementary file 3 — Supplementary Fig. 2 [file 41418_2022_931_MOESM3_ESM.tif]

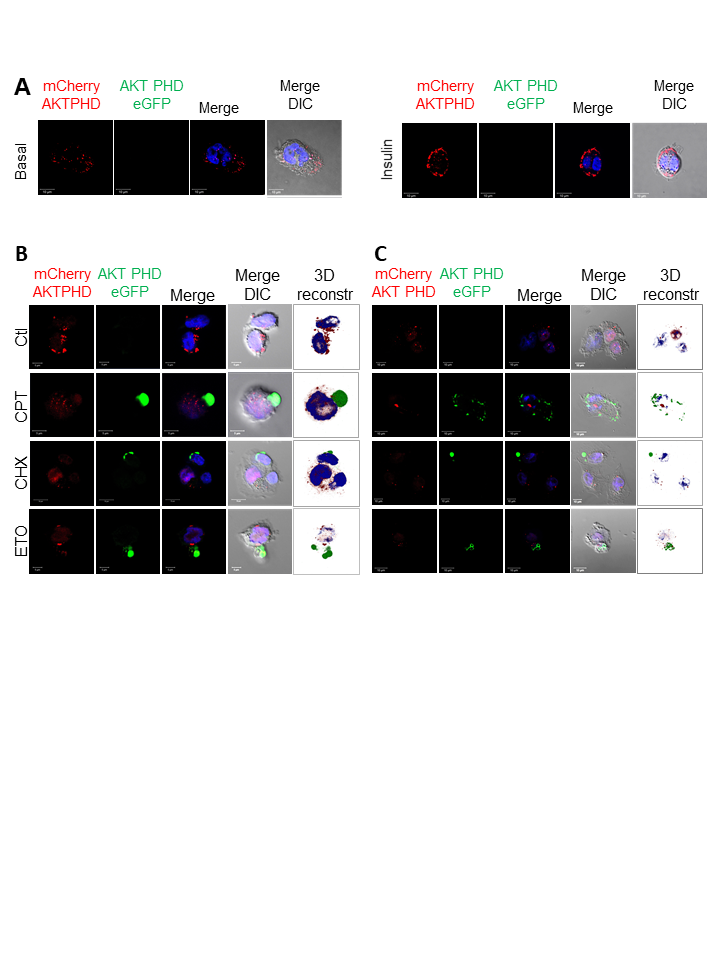

Supplement: Supplementary file 4 — Supplementary Fig.3 [file 41418_2022_931_MOESM4_ESM.tif]

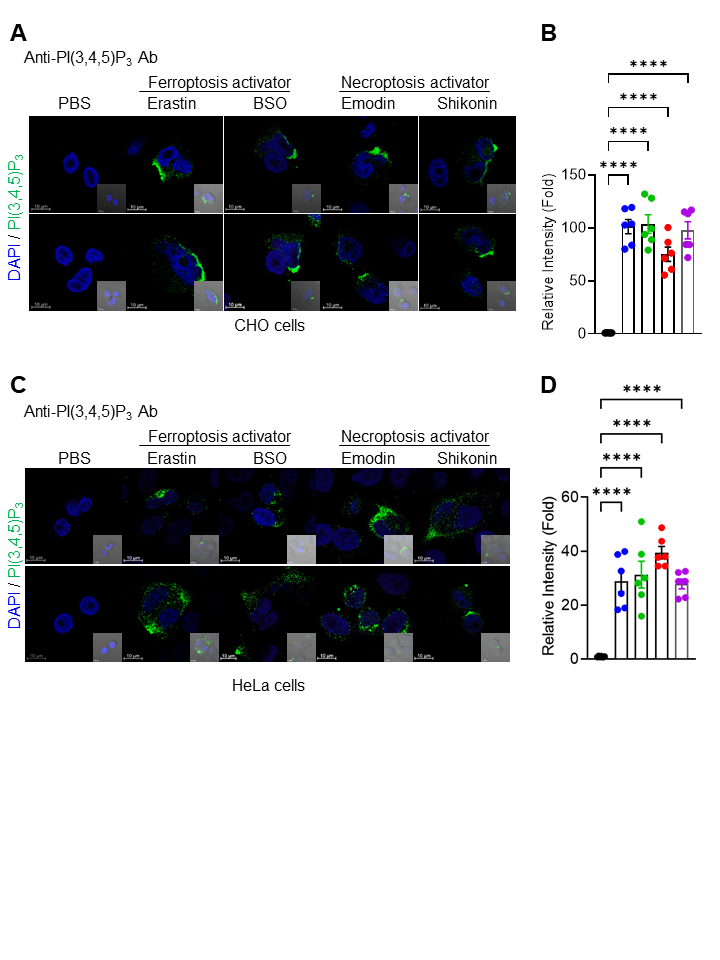

Supplement: Supplementary file 5 — Supplementary Fig.4 [file 41418_2022_931_MOESM5_ESM.tif]

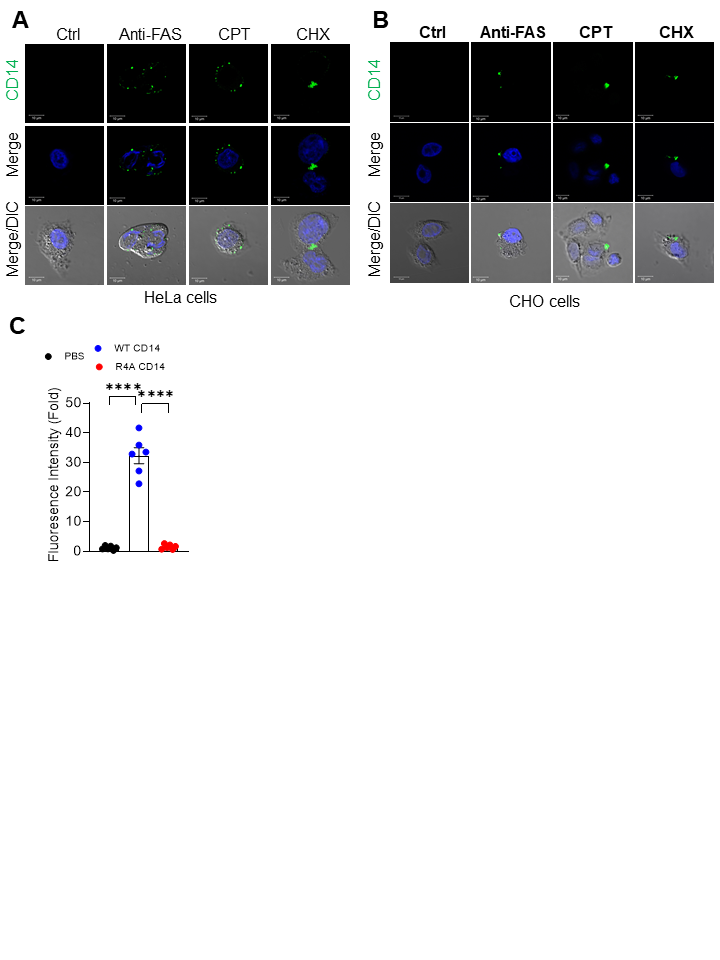

Supplement: Supplementary file 6 — Supplementary Fig.5 [file 41418_2022_931_MOESM6_ESM.tif]

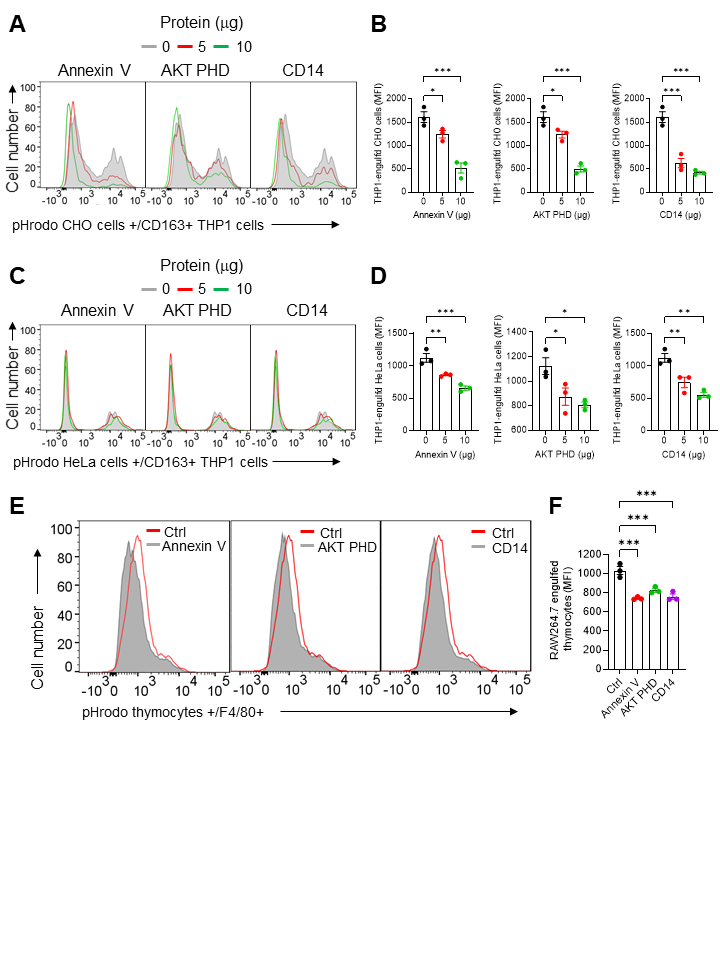

Supplement: Supplementary file 7 — Supplementary Fig.6 [file 41418_2022_931_MOESM7_ESM.tif]

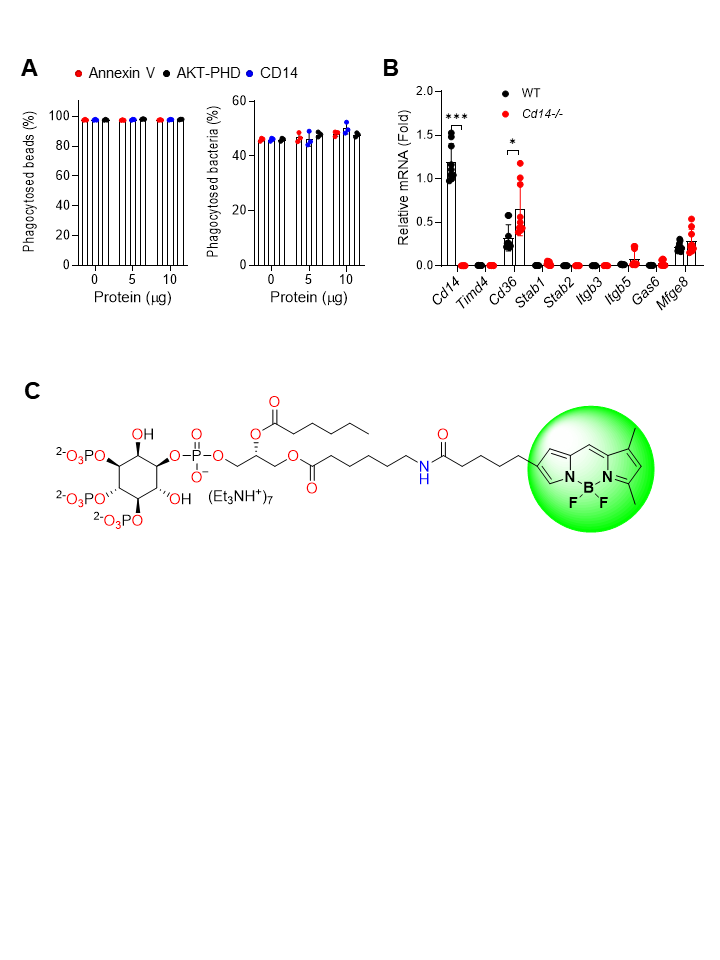

Supplement: Supplementary file 8 — Supplementary Fig.7 [file 41418_2022_931_MOESM8_ESM.tif]

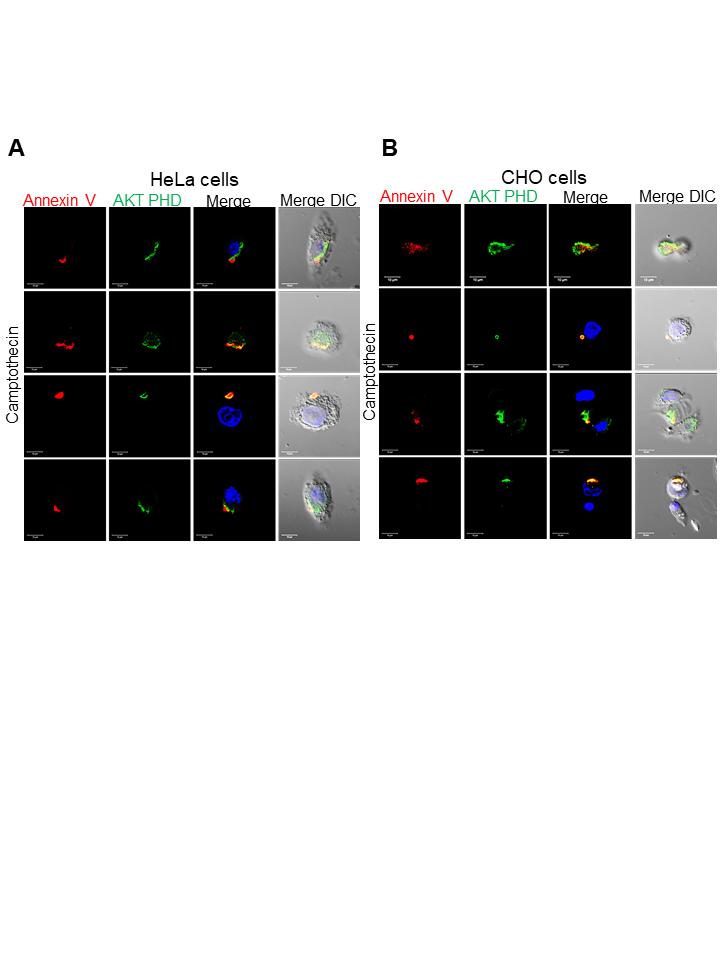

Supplement: Supplementary file 9 — Supplementary Fig. 8 [file 41418_2022_931_MOESM9_ESM.tif]

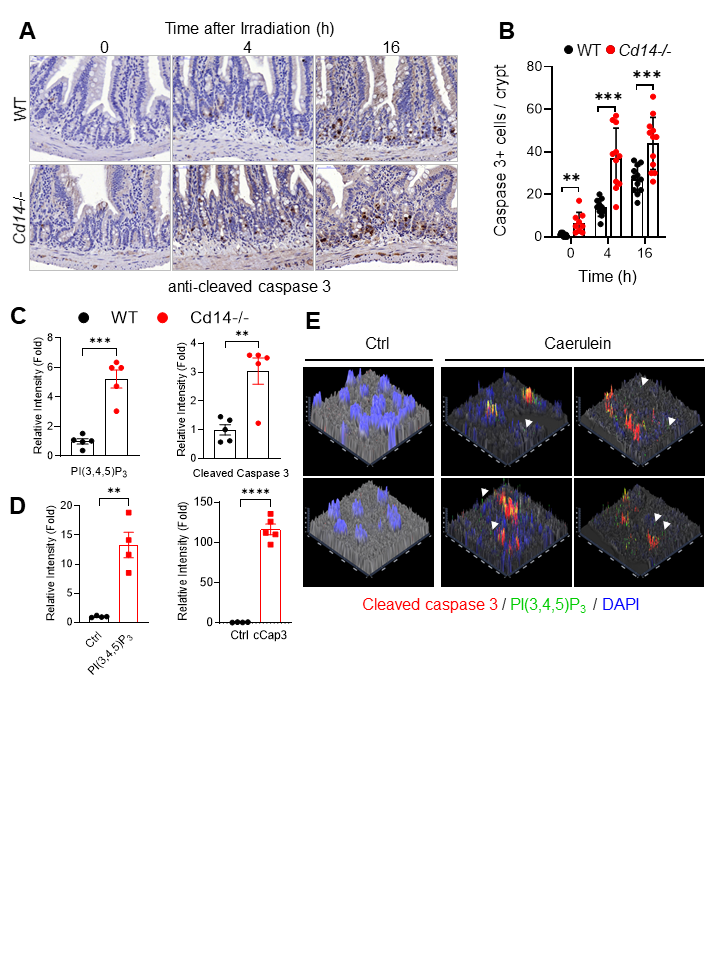

Supplement: Supplementary file 10 — Supplementary Fig.9 [file 41418_2022_931_MOESM10_ESM.tif]
