# Supplementary material for: Externalized phosphatidylinositides on apoptotic cells are eat-me signals recognized by CD14
Source: Cell Death Differ. 2022 Jan 11;29(7):1423–32. doi: 10.1038/s41418-022-00931-2 (PMC9287416; doi:10.1038/s41418-022-00931-2)
Supplement: Supplementary file 12 — Supplementary information [file 41418_2022_931_MOESM12_ESM.docx]

***Supplementary Materials and Methods***

***Data reporting.*** As we used homogeneous cell lines or inbred mice for this study, sample sizes for all experiments were based on preliminary results and previous experience of conducting related experiments. Power calculations were not used to determine sample sizes. The animals for each group of experiments were randomly assigned. No animals were excluded from statistical analyses.

***Cell lines*.** Cell lines obtained from ATCC were authenticated by Short Tandem Repeats (Jurkat, HeLa, and HEK 293T) or interspecies analyses (CHO-K1, RAW264.7) and were mycoplasma free.

***Cell culture conditions.*** HEK293, RAW264.7, THP1, and HeLa cells were grown in DMEM medium (Welgene, Korea) supplemented with 10% fetal bovine serum (FBS) and 1 × penicillin-streptomycin. Jurkat cells and CHO-K1 and CHO-IR/IRS1 cells were grown in RPMI or F12 medium, respectively, supplemented with 10% FBS and 1 × penicillin-streptomycin. At 70%–80% confluency, transient transfection in HEK293T or CHO-K1 cells was performed using Lipofectamine 2000 (Invitrogen) with total DNA of 1 or 10 μg. Cells were maintained at 37°C under 5% CO_2_.

***In vivo phagocytosis***. To assay the ability of peritoneal macrophages to engulf apoptotic cells *in vivo* using a modified method as described by Hu et al. [1], we initially irradiated C57BL/6J mice to induce apoptosis in the thymus. At 16 h after irradiation, the thymus was gently disrupted and filtered through nylon mesh to remove aggregates [2]. Thymocytes were then resuspended with Red Blood Cell lysing buffer (R7757, Sigma-Aldrich) for 15 min to lyse erythrocytes, labeled with pHrodo (5 μmol, 30 min; P35372, Life Technologies), and washed with live-cell imaging solution (A14291DJ, Life Technologies). Next, 8 × 10^6^ pHrodo-labeled thymocytes in 200 μl PBS buffer were injected into the peritoneal cavity of WT C57BL/6J or Cd14-/- mice. Peritoneal lavages were collected 15 min after injection and peritoneal cells were stained with APC-labeled F4/80 antibody. F4/80+/pHrodo+ cells were analyzed by flow cytometry to determine peritoneal macrophage-mediated phagocytosis of pHrodo-labeled apoptotic thymocytes.

***Antibodies.*** The following antibodies were used for western blotting, confocal microscopy, and flow cytometry: hCD14 (NB100-2807, Novus), mCD14 (HPA002127, Atlas Antibodies), mouse F4/80 (BM8, eBioscience), β-actin (AC-15, Sigma-Aldrich), hAKT PHD (05-591, Millipore), hAnnexin V (ab14196, Abcam), cleaved caspase 3 (D175, Cell Signaling Technology), hFAS (EOS9.1, BioLegend) PI(4,5)P_2_ (Life Technologies, A21327), hCD163 (ab182422, Abcam) and PI(3,4,5)P_3_ (Z-P345b, Echelon).

***IC-IP_6_ uptake and binding proteins.*** The livers of C57BL/6 mice harvested 4 h after intravenous administration of IC-IP_6_ (10 mg/kg) were fixed in formalin and embedded in paraffin. As described previously [3], TPMs obtained by peritoneal lavage of C57BL/6 mice 3–5 days after injection of Brewer thioglycolate medium (Difco) were plated on coverslips (10^5^ cells/ml). Iron uptake was visualized using PB (10% potassium ferrocyanide, 25% HCl) or quantified spectrophotometrically (AU480 Chemistry System, Beckman Coulter). To identify binding proteins, IC-IP_6_ was added to RAW264.7 cell lysates (1 mM, 30 min). The precipitated proteins were isolated by centrifugation, separated by SDS-PAGE, trypsin-digested, and identified by LC-MS/MS analysis (Agilent 1260 HPLC, 6550 Quadrupole Time-of-Flight Mass Spectrometer)[.](#_ENREF_44)

***Lipid nanoparticle-protein binding assay***. To determine whether CD14 binds to PI(4,5)P_2_ and PI(3,4,5)P_3_, we used custom-made lipid nanoparticles (Echelon Biosciences) with lipid coating at a molar ratio of PC:PE (95:5), PC:PE:PS (75:5:20), PC:PE:PS:PI(4,5)P_2_ (65:5:20:10), and PC:PE:PS:PI(3,4,5)P_3_ (70:5:20:5). Flow cytometry analyses of the binding properties of CD14, AKT PHD, or anti-PIP3 antibodies for custom-made lipid nanoparticles were performed according to the manufacturer's instructions. Lipid nanoparticles were blocked in 3% fatty acid-free bovine serum albumin (Sigma-Aldrich) in washing/binding buffer (10 mM HEPES, pH 7.4, 150 mM NaCl, and 0.25% NP40) at 4 °C for 1 h. Blocked lipid nanoparticles were incubated with 5 μg of Alexa 647-conjugated CD14, AKT PHD anti-PIPS antibody, or control IgG antibody at 4 °C for 1 h, washed three times with washing buffer, and Alexa 647-conjugated protein or antibody binding to lipid nanoparticles were detected by flow cytometry.

***Protein expression and phospholipid binding specificity.*** Human *CD14* cDNA was subcloned into a pcDNA6 vector for expression with C-terminal V5 and His_6_ tags. Site-specific mutagenesis was performed using a QuikChange Site-Directed Mutagenesis Kit (Stratagene), and sequences were verified. The cDNA encoding hAKT PHD (residues 1–144) was subcloned into the pmCherry-C1 vector (TaKaRa Clontech). CHO cells were transfected using Lipofectamine (Life Technologies) and selected using blasticidin. Cells positive for mCherry were sorted by flow cytometry. The cDNAs encoding the hAKT PHD (1–144), full-length hAnnexin V, and hAKT PHD/eGFP fusion proteins were subcloned into the pET28a vector (Novagene); recombinant proteins with C-terminal His_6_ tags were expressed in *E*. *coli* and purified using Ni-NTA resin and gel filtration. Immobilised phospholipids (P-6001 PIP strips and P-6100 PIP arrays, Echelon Bioscience) were used to assess binding specificity of recombinant CD14, AKT PHD, and Annexin V according to the manufacturer's instructions.

***Isothermal titration calorimetry.*** Phospholipid-related ligands were titrated into protein solutions in 10 mM HEPES, pH 7.0 at 25°C using a microcalorimetry system (iTC200, MicroCal); curves were fit using Origin software (MicroCal). As described previously [4], liposomes were generated by mixing chloroform solutions of 1-palmitoyl-2-oleoyl-sn-glycero-3-phosphocholine (POPC) with 1,2-dipalmotoyl-sn-glycero-3-phosphatidylinositol-3,4,5-trisphosphate (PI(3,4,5)P_3_, Cayman Chemicals) at 80:20 molar ratios. Organic solvents were removed, and phospholipids were vortexed to produce unilamellar vesicles in HEPES, pH 7.0.

***Immunohistochemistry (IHC).*** Tissues were fixed in 10% neutral buffered formalin and embedded in paraffin. To confirm the presence of IC-IP_6_ in the cytoplasm of macrophages, we stained sections (2.5 μm for lymph nodes, and 5 μm for other tissues) with PB and IHC staining with either a rat anti-mouse monoclonal F4/80 (clone BM8, eBioscience) or a rat anti-mouse monoclonal cleaved caspase 3 (clone D175, Cell Signaling Technology) antibody. For liver sections, we used a horseradish peroxidase-conjugated secondary antibody to F4/80 (anti-rat IgG) or to cleaved caspase 3 (anti-rabbit IgG) with a 3,3,-diamino-benzidine tetrahydrochloride substrate (DAKO), and then stained with PB as described above. For breast tumour and lymph node sections, we stained with PB, obtained section images, and performed IHC staining on the same sections stained with PB.

**Supplementary Fig. 1. The role of CD14+ macrophages in IC-IP6 uptake and PIP recognition**. (**A)** IC-IP_6_ was prepared by combining equimolar (10 mM) amounts of IP_6_, FeCl_3_, and CaCl_2_ at pH 6.0. (**B**) Mass spectrometric identification of CD14 (**Fig. 1D**, black arrowhead). Peptides eluted with trypsin were identified by LC-MS/MS. (**C**,**D)** qRT-PCR results for TPMs treated either with LPS (**C**) or using a humidified hypoxic chamber (**D**) for 24 h (Cd14 relative to CypA). (**E)** Quantification of iron-stained with 2,4,6-Tri-(2-pyridyl)-5-triazine (TPTZ) from liver lysates of IC-IP_6_-treated WT and Cd14-/- mice. (**F)** Schematic structures of PI(3,4,5)P_3_, with DAG referring to the diacylglycerol moiety of each phospholipid. (**G**) The mean fluorescence intensity of Ctrl antibody, CD14, AKT PHD, and anti-PIP3 antibody binding to silica particles loaded with specific combinations of phospholipids. All comparisons were performed using a one-way ANOVA with Tukey’s post-hoc multiple comparison test. All data are presented as mean ± standard deviation (SD) for each group (n = 3–8 per group), n = 3 biologically independent replicates. *p < 0.05, **p < 0.01, ***p < 0.001 compared to controls. (**H**) ITC results for 80% PC/20% PI(3,4,5)P3 liposomes titration into 30 μM CD14; Kd values were determined using Origin software (MicroCal). (**I**) Schematic representation of purified CD14 mutant proteins against varying concentrations of phosphatidylinositol phosphates (PIP arrays, Echelon Biosciences)

**Supplementary Fig. 2. Visualizing externalized PIPs on apoptotic cells.** (A-B) CHO cells were treated 6 h with camptothecin (10 μM), cycloheximide (100 μM), or etoposide (100 μM) to induce apoptosis. Cells were stained using anti-PI(3,4,5)P_3_ antibody or recombinant AKT PH domain/anti-AKT PH domain antibody (1:1,000, Millipore) followed by FITC-labeled secondary antibody and DAPI. Differential interference contrast microscopy (DIC; scale bars, 10 μm). (C) Fluorescence intensity of externalized PIPs on the surface of apoptotic cells was quantified using ImageJ software. All comparisons were analyzed by one-way ANOVA with Tukey's post-hoc multiple comparison test. All data are presented as mean ± standard deviation (SD) for each group (n = 4-6 per group), n = 3 biologically independent replicates. *p < 0.05, **p < 0.01, ***p < 0.001, ****p < 0.0001 compared to controls. (D) Cells were stained using anti-PI(4,5)P_2_ antibody followed by FITC-labeled secondary antibody and DAPI. Differential interference contrast microscopy (DIC; scale bars, 10 μm).

**Supplementary Fig. 3**. **Visualizing externalized PIPs on apoptotic cells**. (**A–C**) CHO cells stably expressing the insulin receptor, IRS-1, and an mCherry/AKT PHD fusion protein were used to distinguish intracellular vs. externalized PI(3,4)P_2_ and PI(3,4,5)P_3_. Intracellular PIPs were visualized with the stably expressed mCherry/AKT PHD, whereas externalized PI(3,4,5)P3 was visualized with recombinant eGFP/AKT PHD fusion protein. (**A**) CHO cells were treated with or without. insulin (100 ng/ml, 10 min). (**B** and **C**) CHO cells were treated with camptothecin (CPT, 10 μM), cycloheximide (CHX, 100 μM), or etoposide (ETO, 100 μM) for 6 h. duplicate examples are meant to give a sense of the variability in exofacial display of PIPs (all scale bars, 10 μm). DIC: differential interference contrast. Three-dimensional reconstruction images were made using Zeiss Zen SP2 software.

**Supplementary Fig. 4. Visualizing externalized PIPs on ferroptotsis and necroptosis cells.** (A) CHO cells were treated for 3 h with Erastin (1 μM), L-Buthionine-sulfoximine (BSO, 1 μM), Emodin (5 μM) or Shikonin (1 μM) to induce ferroptosis or necroptosis. Cells were stained using anti-PI(3,4,5)P3 antibody followed by FITC-labeled secondary antibody and DAPI. Differential interference contrast microscopy (DIC; scale bars, 10 μm). (B) The fluorescence intensity of externalized PIPs on the surface of ferroptotsis and necroptosis cells was quantified using ImageJ software. (C) HeLa cells were treated 3 h with Erastin (1 μM), L-Buthionine-sulfoximine (BSO, 1 μM), Emodin (5 μM) or Shikonin (1 μM) to induce ferroptosis or necroptosis. Cells were stained using anti-PI(3,4,5)P3 antibody followed by FITC-labeled secondary antibody and DAPI. Differential interference contrast microscopy (DIC; scale bars, 10 μm). (D) The fluorescence intensity of externalized PIPs on the surface of ferroptotsis and necroptosis cells was quantified using ImageJ software. All comparisons were performed by one-way ANOVA with Tukey's post-hoc multiple comparison test. All data are presented as mean ± standard deviation (SD) for each group (n = 6 per group), n = 3 biologically independent replicates. ****p < 0.05, **p < 0.01, ***p < 0.001 compared to controls.

**Supplementary Fig. 5 PIP externalization and its role in phagocytosis**. (**A**) HeLa or (**B**) CHO cells treated with anti-FAS antibody (150 ng/ml), camptothecin (CPT, 10 μM) or cycloheximide (CHX, 100 μM) for 6 hr were stained with 5 μg CD1, an anti-CD14 antibody (1:1,000, Sigma), an FITC-labeled secondary antibody, and DAPI (scale bars, 10 μm). (**C**) The fluorescence intensity of externalized PIPs on the surface of apoptotic cells was quantified using ImageJ software. All comparisons were performed by one-way ANOVA with Tukey's post-hoc multiple comparison test. All data are presented as mean ± standard deviation (SD) for each group (n = 6 per group). n = 3 biologically independent replicates. *p < 0.05, **p < 0.01, ***p < 0.001 compared to controls.

**Supplementary Fig. 6. *In vitro* phagocytosis of apoptotic cells. (A-B),** Representative flow cytometric plots (**A**, left) and MFI values (**B**, right) of PMA/LPS-stimulated CD163+ THP1 phagocytosis of actinomycin D (0.1μg/ml)-treated, pHrodo-labeled CHO cells using recombinant Annexin V, AKT PHD, and CD14 proteins to mask externalized PS or PIPs. Actinomycin D-treated CHO cells were labeled with pHrodo for 30 min prior to adding the PMA/LPS-stimulated CD163+ THP1 cells for 1 h. **(C-D),** Representative flow cytometric plots (**C**, left) and MFI values (**D**, right) of PMA/LPS-stimulated CD163+ THP1 phagocytosis of actinomycin D (0.1μg/ml)-treated, pHrodo-labeled HeLa cells using recombinant Annexin V, AKT PHD, and CD14 proteins to mask externalized PS or PIPs. Actinomycin D-treated HeLa cells were labeled with pHrodo for 30 min prior to adding the PMA/LPS-stimulated CD163+ THP1 cells for 1 h. (**E,F)**, Representative flow cytometric plots (**E,** left) and MFI values (**F,** right) of LPS-stimulated RAW264.7 phagocytosis of irradiated thymocytes using recombinant Annexin V, AKT PHD, and CD14 proteins to mask externalized PS or PIPs. Thymocytes were labeled with pHrodo for 30 min prior to adding LPS-stimulated RAW264.7 cells for 1 h. All comparisons were performed by one-way ANOVA with Tukey's post-hoc multiple comparison test. All data are presented as mean ± standard deviation (SD) for each group (n =3 per group), n = 3 biologically independent replicates. ****p < 0.05, **p < 0.01, ***p < 0.001 compared to controls.

**Supplementary Fig. 7.** **The effects of Annecxin V, AKT PHD, and CD14 on phagocytosis.** (**A**) Phagocytosis of FITC-labeled latex beads (left) and pHrodo-labeled E. coli (right) by TPMs were incubated with Annexin V, AKT PHD, or CD14. (**B**) Relative mRNA levels of various eat-me receptors in TPMs treated with LPS (0.5 μg/ml for 16 h) from WT and Cd14-/- mice (mean ± SD, n = 6). (**C**) Schematic structures of a synthetic Bodipy-labeled PI(3,4,5)P_3_ (Echelon Biosciences).

**Supplementary Fig.8. Both AKT PHD and Annexin V proteins recognize apoptotic cells**. After inducing apoptosis with camptothecin (10 μM), (**A**) HeLa and (**B**) CHO cells were treated with recombinant AKT PHD or Annexin V for 30 min followed by appropriate primary and FITC- or Texas Red-labeled secondary antibodies, respectively. DIC, differential interference contrast microscopy.

**Supplementary Fig. 9. In vivo induction of exofacial PIPs.** **Supplementary Fig. 9. In vivo induction of exofacial PIPs.** (**A**) Representative images of jejunum sections from irradiated WT and Cd14-/- mice were stained for cleaved caspase 3. (**B**) Cleaved caspase 3-positive cells were quantified within 8–10 low-power fields (mean ± SD, n = 5–10). Scale bars, 50 μm. All comparisons were performed by one-way ANOVA with Tukey’s post-hoc multiple comparison test. All data are presented as mean ± standard deviation (SD) for each group (n = 3, 9–12 lpf/mouse). *p < 0.05, **p < 0.01, ***p < 0.001 compared to controls. (**C**) The fluorescence intensity of externalized PIPs (left) on the surface of apoptotic cells and cleaved caspase 3 (right) in the jejunum sections from irradiated WT and Cd14-/- mice were quantified using ImageJ software. All comparisons were performed by unpaired student t-test. All data are presented as mean ± standard deviation (SD) for n = 5 biologically independent replicates. **p < 0.01, ***p < 0.001 compared to controls. (**D**) The fluorescence intensity of externalized PIPs (left) on the surface of apoptotic cells and cleaved caspase 3 (right) in the pancreatic sections from mice treated with or without caerulein was quantified using ImageJ software. (E) 2.5 D confocal images of pancreas tissue sections. Cleaved caspase 3^+^ cells PI(3,4,5)P_3_^+^ or double-positive cells were quantified from stained tissue sections. All comparisons were performed by unpaired student *t*-test. All data are presented as mean ± standard deviation (SD) for each group (n=5-6 per group), n = 3 biologically independent replicates per group. **p < 0.01, ***p < 0.001 compared to controls.

**Supplementary movie 1**. **Representative video of externalized PI(3,4)P_2_ and PI(3,4,5)P_3_ detection using a recombinant eGFP/AKT PHD fusion protein on CHO cells expressing mCherry/AKT PHD fusion protein**. Apoptosis was induced in CHO cells that stably expressed mCherry/AKT PHD fusion protein to visualize intracellular PI(3,4)P_2_ and PI(3,4,5)P_3_, while externalized PIPs were visualized using the recombinant eGFP/AKT PHD fusion protein. Differential interference contrast (DIC) microscopy.

**Supplementary movie 2. Representative video of Annexin V and AKT PHD binding of apoptotic cells.** CHO cells treated 6 h with 10 μM camptothecin and incubated with Annexin V and AKT PHD were detected with primary antibodies and Texas Red- or FITC-labeled secondary antibodies, respectively. Differential interference contrast (DIC) microscopy.

**Aditional References**

1. Hu B, Sonstein J, Christensen PJ, Punturieri A, Curtis JL. Deficient In Vitro and In Vivo Phagocytosis of Apoptotic T Cells by Resident Murine Alveolar Macrophages. *The Journal of Immunology* 2000, **165**(4)**:** 2124-2133.

2. Um H-N, Baek J-O, Park S, Lee E-H, Jang J, Park W-J*, et al.* Small intestinal immune-environmental changes induced by oral tolerance inhibit experimental atopic dermatitis. *Cell Death & Disease* 2021, **12**(3)**:** 243.

3. Lee J-H, Phelan P, Shin M, Oh B-C, Han X, Im S-S*, et al.* SREBP-1a–stimulated lipid synthesis is required for macrophage phagocytosis downstream of TLR4-directed mTORC1. *Proceedings of the National Academy of Sciences* 2018, **115**(52)**:** E12228-E12234.

4. Kang JK, Kim OH, Hur J, Yu SH, Lamichhane S, Lee JW*, et al.* Increased intracellular Ca(2+) concentrations prevent membrane localization of PH domains through the formation of Ca(2+)-phosphoinositides. *Proc Natl Acad Sci U S A* 2017, **114**(45)**:** 11926-11931.
