# Supplementary material for: Externalized phosphatidylinositides on apoptotic cells are eat-me signals recognized by CD14
Source: Cell Death Differ. 2022 Jan 11;29(7):1423–32. doi: 10.1038/s41418-022-00931-2 (PMC9287416; doi:10.1038/s41418-022-00931-2)
Supplement: Supplementary file 13 — Representative video of externalized PI(3,4)P2 and PI(3,4,5)P3 detection using a recombinant eGFP/AKT PHD fusion protein on CHO cells expressing mCherry/AKT PHD fusion protein. [file 41418_2022_931_MOESM13_ESM.pptx]

## Slide 1
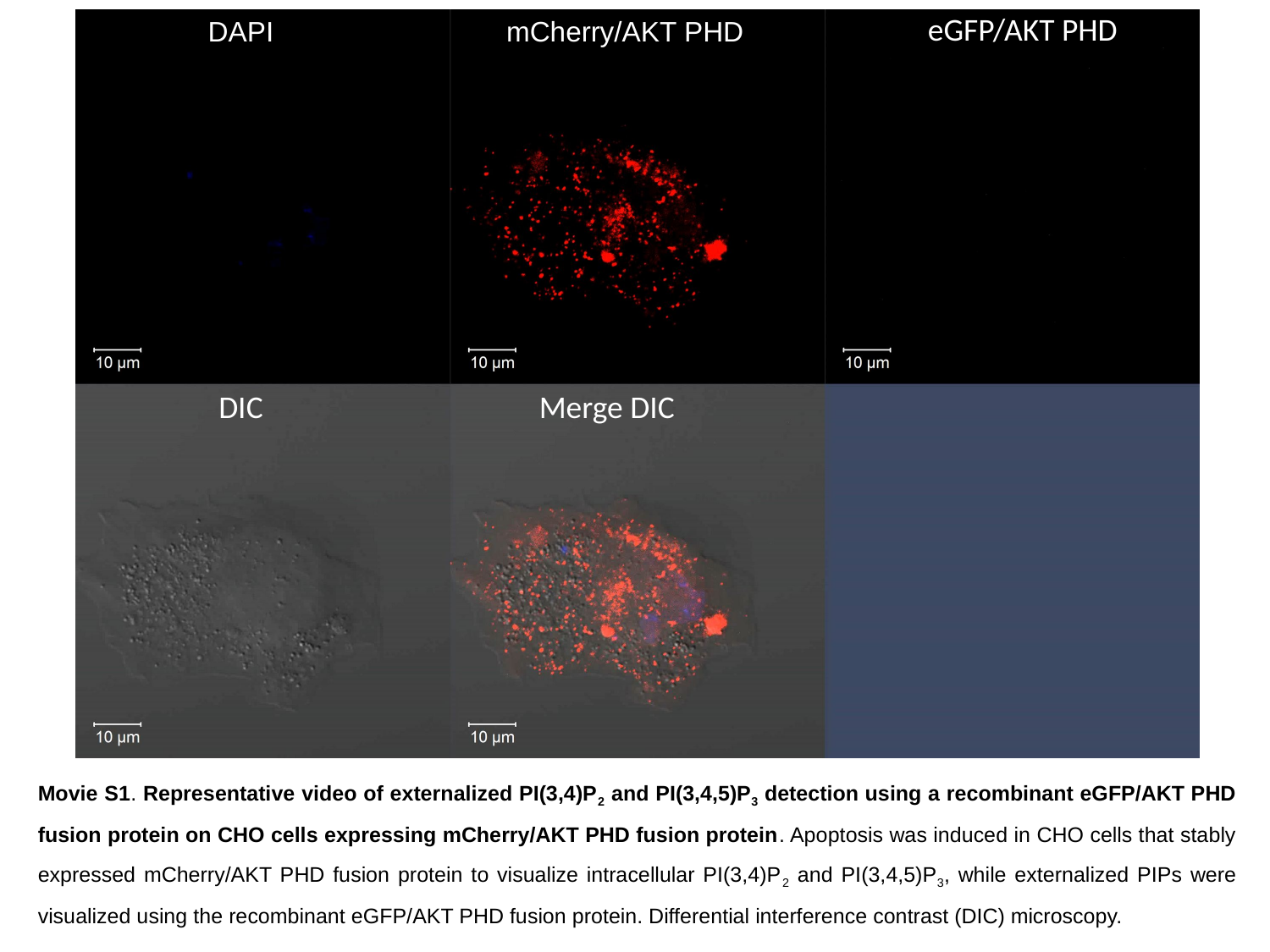

eGFP/AKT PHD
DAPI
mCherry/AKT PHD
DIC
Merge DIC
Movie S1. Representative video of externalized PI(3,4)P2 and PI(3,4,5)P3 detection using a recombinant eGFP/AKT PHD fusion protein on CHO cells expressing mCherry/AKT PHD fusion protein. Apoptosis was induced in CHO cells that stably expressed mCherry/AKT PHD fusion protein to visualize intracellular PI(3,4)P2 and PI(3,4,5)P3, while externalized PIPs were visualized using the recombinant eGFP/AKT PHD fusion protein. Differential interference contrast (DIC) microscopy.
