# Supplementary material for: Externalized phosphatidylinositides on apoptotic cells are eat-me signals recognized by CD14
Source: Cell Death Differ. 2022 Jan 11;29(7):1423–32. doi: 10.1038/s41418-022-00931-2 (PMC9287416; doi:10.1038/s41418-022-00931-2)
Supplement: Supplementary file 14 — Supplementary movie 2. Representative video of Annexin V and AKT PHD binding of apoptotic cells. [file 41418_2022_931_MOESM14_ESM.pptx]

## Slide 1
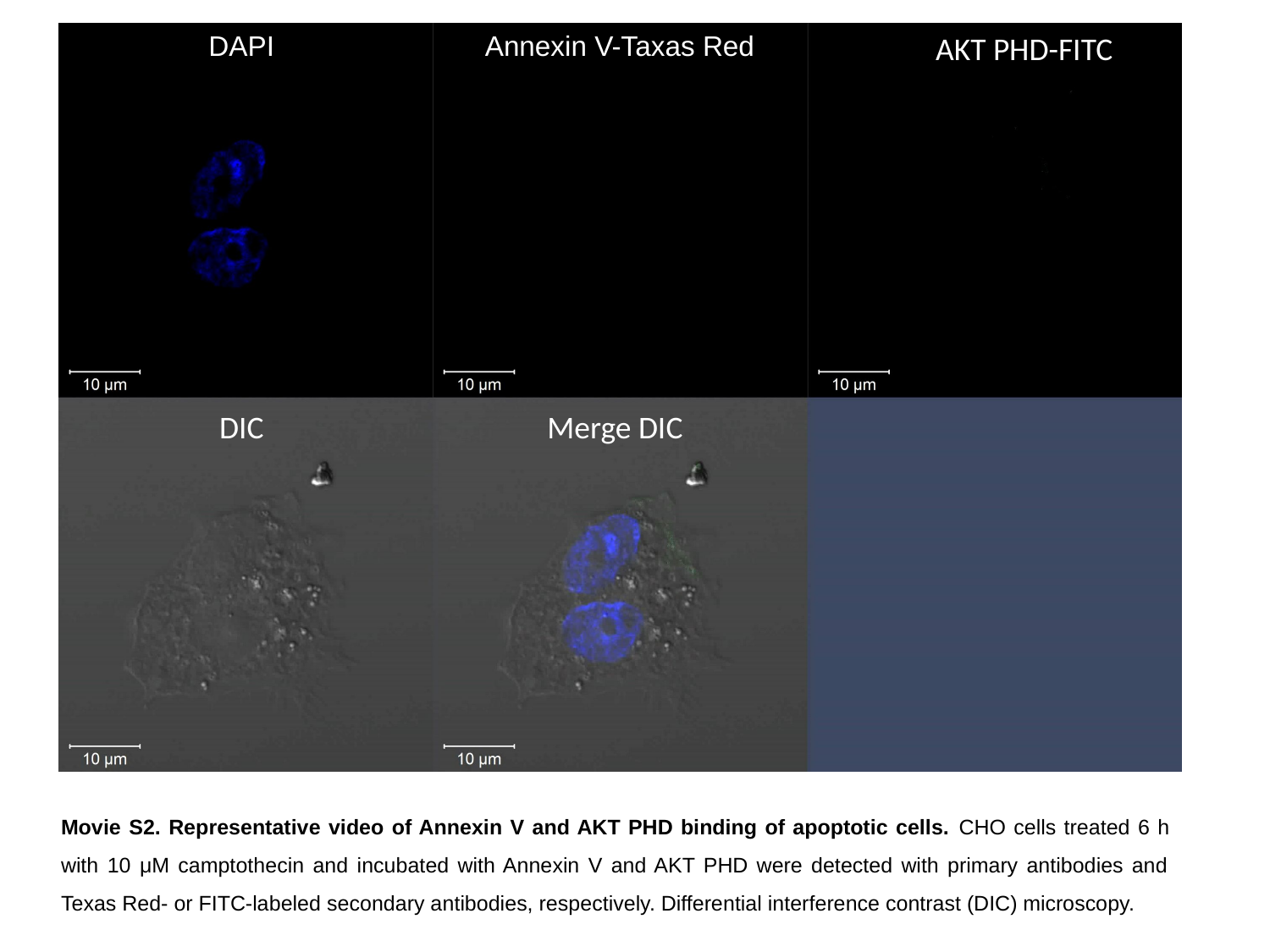

DAPI
Annexin V-Taxas Red
AKT PHD-FITC
DIC
Merge DIC
Movie S2. Representative video of Annexin V and AKT PHD binding of apoptotic cells. CHO cells treated 6 h with 10 μM camptothecin and incubated with Annexin V and AKT PHD were detected with primary antibodies and Texas Red- or FITC-labeled secondary antibodies, respectively. Differential interference contrast (DIC) microscopy.
